# Estimated Burden of Influenza and Direct and Indirect Benefits of Influenza Vaccination

**DOI:** 10.1001/jamanetworkopen.2025.21324

**Published:** 2025-07-16

**Authors:** Mary G. Krauland, Alexis Mandell, Mark S. Roberts

**Affiliations:** 1 Department of Health Policy and Management, University of Pittsburgh School of Public Health, Pittsburgh, Pennsylvania; 2 Public Health Dynamics Laboratory, University of Pittsburgh School of Public Health, Pittsburgh, Pennsylvania

## Abstract

**Importance:**

Vaccination is the safest and most effective way to prevent infectious diseases. It not only reduces disease in the vaccinated portion of a population but also potentially provides indirect benefit to the unvaccinated portion. Indirect benefits to the unvaccinated portion in a population are difficult to estimate since indirect benefits cannot be determined from the general population simply.

**Objective:**

To estimate the influenza case burden, both direct and indirect, averted by vaccination using an agent-based model, which models agents as individuals in the simulation population.

**Design, Setting, and Participants:**

In this decision analytical modeling study, simulations included varied levels of virus transmissibility, vaccine effectiveness, and vaccine uptake among the 2010 Allegheny County, Pennsylvania, census population. Simulations were run from August 15, 2022, to May 31, 2023. Individuals were vaccinated beginning in September of the simulation year.

**Exposure:**

Influenza infection.

**Main Outcomes and Measures:**

Differences between number of influenza cases in vaccinated and unvaccinated portions of the population.

**Results:**

The population used in this study consisted of 1 218 695 agents (median [IQR] age, 40.6 [3.6-77.6] years; 51% female) and was statistically similar in demographics to the 2010 Allegheny County census population. The mean (SD) burden of influenza averted by vaccination with a vaccine effectiveness of 40% ranged from 32.9% (0.9%) in the high transmission scenario to 41.5% (3.4%) in the low transmission scenario for seasonal influenza. In the model, indirect benefit to the unvaccinated portion of the population was found over a range of modeled transmissibility characteristics of seasonal influenza and varied levels of vaccine effectiveness and vaccination coverage; however, direct benefit to the vaccinated portion was greater in all scenarios. At the highest levels of transmissibility, such as might be found in pandemic influenza, indirect benefit decreased and eventually disappeared.

**Conclusions and Relevance:**

In this analytical model study, influenza vaccination provided substantial benefit in reducing infections to both the vaccinated and unvaccinated portions of the population. Even when both vaccine effectiveness and vaccine uptake were low, vaccination showed marked reductions in disease burden for transmission levels characteristic of seasonal influenza. However, when the level of transmission was very high, even a highly effective vaccine did not protect unvaccinated individuals. These findings underscore the importance of vaccination in disease prevention and control and show that indirect benefits are limited in high transmission situations.

## Key Points

**Question:**

What is the estimated influenza case burden, both direct and indirect, averted by vaccination?

**Findings:**

This decision analytical modeling study in 1 218 695 individuals estimated that the burden of influenza averted by vaccination ranged from 32.9% to 41.5% for seasonal influenza when modeled with a vaccine effectiveness of 40%. For viral strains similar to ones circulating during seasonal influenza, vaccination provided indirect benefit to unvaccinated individuals, but the direct benefit to vaccinated individuals was always greater.

**Meaning:**

These findings suggest that influenza vaccination benefits both the vaccinated and unvaccinated segments of the population and that vaccine effectiveness estimates in those groups may underestimate the actual effectiveness of vaccination.

## Teaser

This decision analytical modeling study estimates the direct and indirect influenza case burden averted by vaccination in a representative US population.

## Introduction

Vaccination stimulates an immune response that directly protects the individual receiving the vaccine from infection by a given pathogen. Vaccination also provides indirect benefit, effectively shielding unvaccinated portions of the population from infection by decreasing the number of exposures unvaccinated individuals receive. Both direct and indirect benefits decrease the number of infections required to reduce the effective reproduction rate to less than 1 and halt transmission. Indirect protection influences the attack rate (AR) of a pathogen in a population since the AR includes both direct and indirect effects. Additionally, since vaccine effectiveness is often calculated as a difference in AR in vaccinated vs unvaccinated individuals, indirect benefits of vaccination may impact the reliability of that calculation.^1^ Quantification of the indirect benefit of vaccination is rarely available from surveillance data, although it can be estimated in some cases.^2^ The indirect benefit of vaccination of one subgroup, typically children, to other unvaccinated subgroups in the population has been documented.^3,4,5,6^ Studies of transmission in groups with mixed vaccination status, such as households and prisons, have provided data that support substantial indirect benefits of vaccination.^7,8^

A number of modeling studies have estimated the indirect protection provided by vaccination using a variety of methods.^9,10,11^ While most studies estimated that indirect benefits exist, there has been no consensus on the level of those benefits.^11^ Due to its ability to directly track vaccination and infection status of individuals (ie, agents in the simulation), our modeling methodology provides novel insights into the complex association between viral transmissibility and the benefits of vaccination, both direct and indirect.

To contribute to understanding population outcomes of vaccination, including overall burden averted and benefits to both the vaccinated and unvaccinated portions of the population, we modeled influenza in an agent-based model capable of following infection in both vaccinated and unvaccinated individuals of the population. The model spreads infection through individual interactions in a number of group locations. We modeled infection with varied levels of transmission, vaccine effectiveness, and vaccine uptake to estimate averted burden and direct and indirect benefits of vaccination under various conditions.

## Methods

In this decision analytical modeling study, we used an agent-based model of influenza implemented in the Framework for Reconstructing Epidemiologic Dynamics (FRED) simulation platform.^12^ The FRED platform uses synthetic populations that are statistically similar at the block group level to census populations. Agents in FRED models have demographic attributes, household locations, and simulated schools and workplaces. These agents spread infections through interactions in mixing groups (eMethods in Supplement 1). The FRED platform has been used previously to model influenza and other respiratory diseases.^13,14,15^ This decision analytical model was approved by the University of Pittsburgh Institutional Review Board. Informed consent was not needed because the study used data from public sources and contained no identifiable information. This study followed the Consolidated Health Economic Evaluation Reporting Standards (CHEERS) guideline.

### Influenza Model

We used the extended Susceptible, Exposed, Infectious, and Recovered model with additional states for presymptomatic and asymptomatic infection (eMethods, eFigure 1, and eTable 1 in Supplement 1). The model has been described previously and includes immunity from prior year infection.^13,16,17^ Reinfections are possible. Simulations were run for a single season (eFigure 2 in Supplement 1) with 100 repetitions (eFigure 3 in Supplement 1).

Influenza case burden is highly variable from season to season. The Centers for Disease Control and Prevention (CDC) estimated that the number of symptomatic disease cases ranged from approximately 9 million to more than 40 million per year from 2010 to 2024 (eMethods and eTable 2 in Supplement 1).^18^ Wide variations in case burden are found even within an influenza subtype in different seasons, presumably due to a number of factors that include, but are not limited to, immunity from prior exposures, antigenic differences between current and earlier strains, and protection provided by vaccination in the current season, which varies by match of vaccine to circulating strain and the level of vaccine uptake. Therefore, for the sake of generalizability, we chose to model with several levels of transmissibility of the influenza virus to produce a range of case burdens.

### Reproductive Rate in FRED

In FRED, reproductive rate (R0 or Rt) is not an input. The R0 of an outbreak is a function of the transmissibility of the pathogen, the characteristics of the disease process (ie, length of latent and infectious periods, presence of asymptomatic transmission), and the characteristics of the population used for simulation (demographics, interaction patterns, and contact rates). Reproductive rate in a simulation is produced as a combination of those factors (eMethods in Supplement 1).^13,14,16^ Since our simulations included immunity from prior influenza infection in a proportion of the population, we report Rt, in the early stages of an outbreak when there is limited population immunity, as a proxy for a true R0.

To account for the wide range in influenza burden, we modeled influenza without vaccination but including prior immunity due to previous exposure to produce approximate Rts of 1.43, 1.81, and 1.88, corresponding to low, moderate, and high seasonal influenza transmission rates, respectively. These Rts correspond to FRED transmissibility parameters of 0.65, 0.70, and 0.75, respectively, in the specific influenza model. We extended this range by adding lower (0.6, approximate Rt = 1.33) and higher (0.80 and 0.85, approximate Rt = 1.96 and 1.98, respectively) levels to investigate the impact of vaccination in those boundary conditions (eTable 3 in Supplement 1). Simulations with no vaccination but including prior immunity were used to produce base ARs for comparisons. We also ran select simulations at higher rates of transmission (ARs of 51.1%, 85.4%, 93.6%, and 96.6% in the absence of vaccination, corresponding to FRED approximate Rt = 2.01, 3.92, 4.99, and 5.04, respectively) to determine whether averted burden followed the same pattern in those cases, which are more similar to pandemic scenarios.

Simulations were performed using a population of Allegheny County, Pennsylvania, which was created from the 2010 population. This population numbered approximately 1.2 million agents and was statistically equivalent to the Allegheny County census population at the block group level. It closely mirrors the US population in demographics, including sex and age, at annual granularity (eTable 4 in Supplement 1). The simulated population includes race as identified in the census but not ethnicity. The population was 12% Black, 80% White, and 8% other races. Neither race nor ethnicity of simulated individuals (agents) contributed to influenza transmission in the simulations because transmission depended on number, length, and intensity of contacts as determined from overall population data. Agents interact in households, which are also realistic at the block group level and are assigned to interaction groups, including schools and workplaces. Agents also interact in neighborhoods, which are 1-km^2^ areas that include their households. The FRED populations and interactions have been previously described.^14^

Immunity from previous year infections was included in the simulation. Reduced susceptibility to infection was distributed to the population randomly using age group–specific rates as reported by the CDC for the 2019 to 2020 season (eMethods and eTable 5 in Supplement 1).

### Vaccination Scenarios

Vaccination at age group–specific rates as reported by the CDC in the simulation population resulted in an overall vaccination rate of approximately 51% in the population (eMethods in Supplement 1). This rate was used for simulations except in scenarios used to investigate the impact of varied levels of vaccination. For those simulations, vaccination rates were increased or decreased in increments of 5% for each age group for minimum and maximum rates of 30% lower to 20% higher, resulting in overall population vaccine uptake rates of 22% to 71% (eTable 6 in Supplement 1). Vaccine effectiveness varies by year, so for most simulations, it was varied from 40% to 60% by 5% increments. Select scenarios included vaccine effectiveness that varied from 30% to 70% by 5% increments. In the model, vaccines were implemented as leaky, with vaccine effectiveness implemented as a reduction in susceptibility to infection.

### Statistical Analysis

#### Main Outcome

For the total population, we calculated AR as total cases of influenza per total population, including asymptomatic and symptomatic cases. For vaccinated (direct benefit) and unvaccinated (indirect benefit) agent AR, we divided the cases in the vaccinated and unvaccinated portions of the population by the number of agents either vaccinated or unvaccinated. We calculated the AR ratio of unvaccinated to vaccinated to use as a measure of the relative direct and indirect impact of vaccination.^11^ Larger values for the AR ratio correspond to smaller indirect effects (less benefit to unvaccinated agents). A ratio of 1 indicates equal benefit to vaccinated and unvaccinated agents.

#### Sensitivity Analyses

We performed several sensitivity analyses to investigate the impact of parameters thought to be of importance in the simulations. Sensitivity analysis results are included in the eMethods, eTables 7 to 9, and eFigures 4 to 8 in Supplement 1. Additionally, we performed select simulation scenarios in other populations that have different demographics to determine whether the results in our main study population are broadly applicable. Overall, while results differed in absolute magnitude for populations with different demographics, the pattern of results were similar in all tested populations (eTables 10-12 in Supplement 1). Statistical significance was not assessed. Analyses were performed using custom Perl scripts and R, version 4.0.3 (R Foundation for Statistical Computing).

## Results

The FRED population used in this study consisted of 1 218 695 agents (median [IQR] age, 40.6 [3.6-77.6] years; 51% female and 49% male). Agent demographics were statistically similar to the 2010 Allegheny County census population.

### Influenza Burden

**Figure 1.**
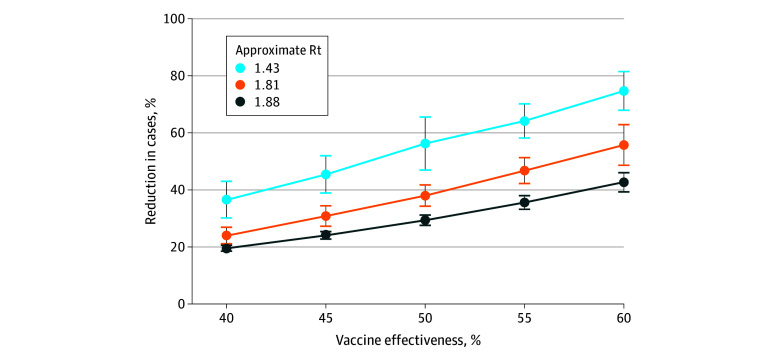
Averted Burden of Influenza Expressed as percent reduction in cases from a nonvaccination scenario by level of transmission and level of vaccine effectiveness. Error bars indicate SD. Rt indicates reproductive rate.

The influenza burden averted by vaccination varied based on the transmissibility of the strain and the effectiveness of the vaccine (Figure 1; eTable 13 in Supplement 1). The mean (SD) reduction in cases in scenarios representative of seasonal influenza ranged from 41.5% (3.4%) to 70.3% (4.0%) in the low transmission scenario (approximate Rt = 1.43), from 34.3% (1.5%) to 56.6% (4.1%) in the moderate transmission scenario (approximate Rt = 1.81), and from 32.9% (0.9%) to 48.1% (1.8%) in the high transmission scenario (approximate Rt = 1.88), depending on the modeled vaccine effectiveness. These findings represent a reduction in total infections, which included both symptomatic and asymptomatic infections. For a lower modeled transmission rate (approximate Rt = 1.33 without vaccination), vaccination showed a mean (SD) reduction from 57.5% (6.0%) to 88.6% (4.2%) in cases. For higher transmission rates, vaccination was still effective at reducing infections, with reduction in burden similar to that found for approximate Rt 1.88 (eTable 13 in Supplement 1).

### Vaccine Uptake

**Figure 2.**
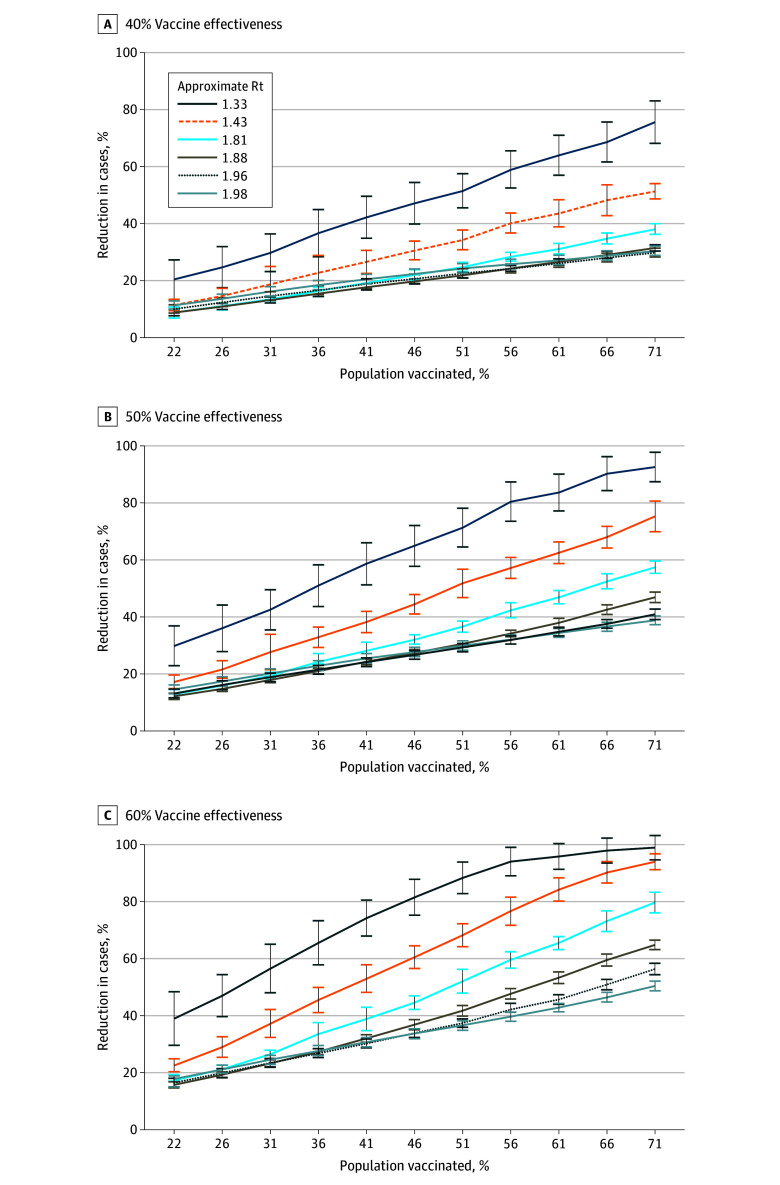
Percentage of Total Influenza Cases Averted Due to Vaccination in Various Transmission Scenarios With Various Vaccine Effectiveness Vaccination in main simulations was by age group at the reported level for 2019 to 2020, which resulted in a 51% vaccination uptake in the population. Decreased and increased vaccination was in decrements or increments of 5% in each age group. Error bars indicate SD. Rt indicates reproductive rate.

The overall reduction in case burden depended on the level of vaccine uptake (Figure 2; eTable 14 in Supplement 1). Reduction in burden was modest when only a small fraction of the population was vaccinated in simulations in which the effectiveness of the vaccine was low, even when transmission was low overall. When vaccine effectiveness was higher (60%), cases could be almost eliminated when only approximately 56% of the population was vaccinated in the lowest transmission scenarios. When transmission was at higher levels, increases in vaccine uptake resulted in less reduction in cases. Vaccine uptake of approximately 61% when the approximate Rt without vaccination was 1.98 still produced a reduction in cases of approximately 50% when vaccine effectiveness was 60%.

### Direct and Indirect Benefits of Vaccination

**Figure 3.**
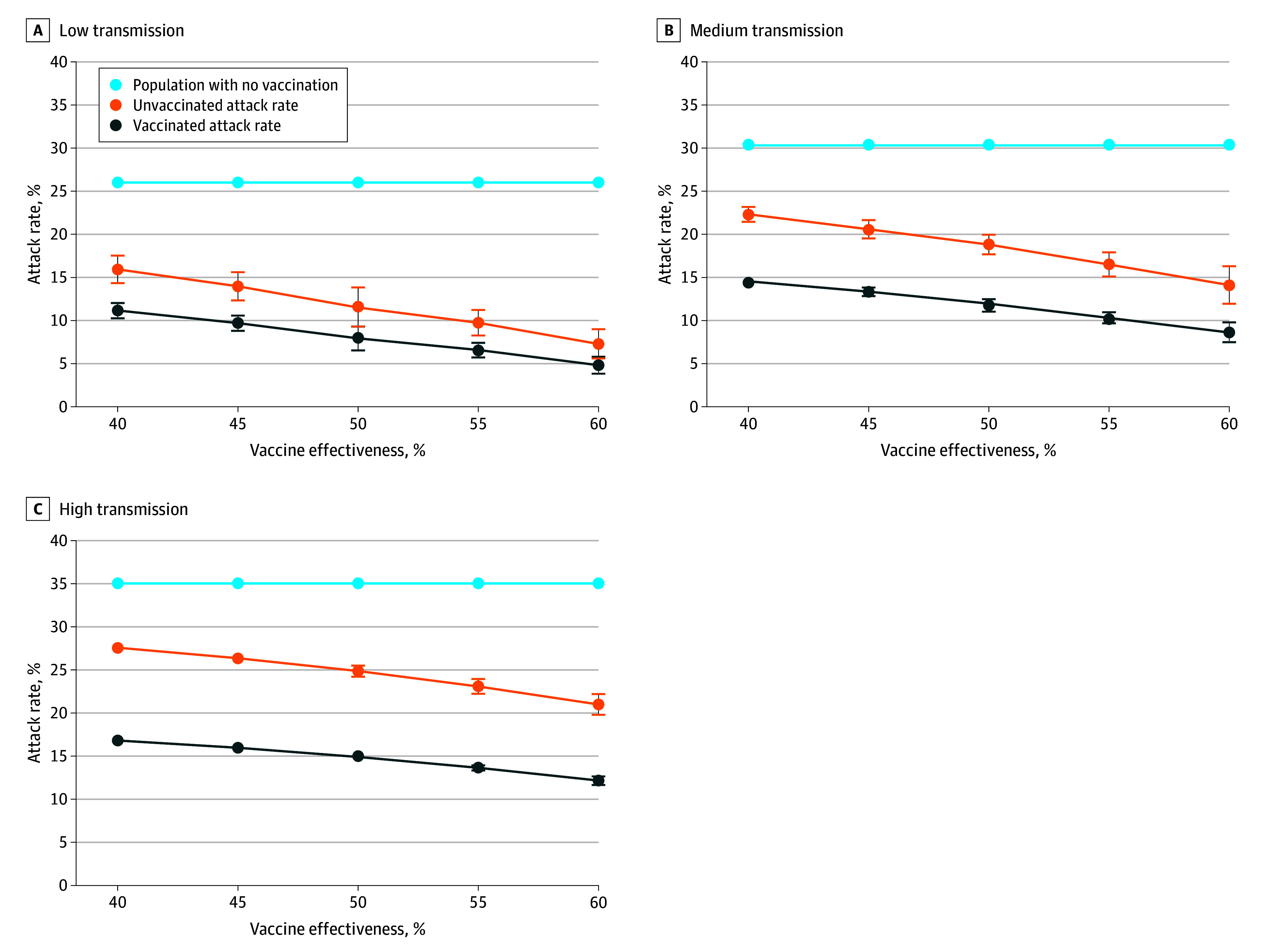
Attack Rate in Vaccinated and Unvaccinated Agents Compared With No Vaccination in Different Transmission Scenarios With Vaccines of Different Levels of Effectiveness While present, error bars, indicating SD, are not always visible.

In scenarios with burden of infection similar to seasonal influenza (approximate Rt = 1.43, 1.81, and 1.88), vaccination reduced case burden in both the vaccinated and unvaccinated portions of the population (Figure 3). In these scenarios, the benefit was higher for the vaccinated portion but still substantial for the unvaccinated portion. The AR ratio of unvaccinated to vaccinated was greater than 1 in all tested combinations of transmissibility and vaccine effectiveness, indicating that the vaccine always provided greater benefit to the vaccinated than the unvaccinated portion (Table). The AR ratio of unvaccinated to vaccinated in scenarios representative of seasonal influenza ranged from 1.43 for the scenario with lowest vaccine effectiveness (40%) and low transmission rate (approximate Rt = 1.43) to 1.73 in the scenario with the highest vaccine effectiveness (60%) and high transmission (approximate Rt = 1.88) (Table). An AR ratio of 1 would indicate that the unvaccinated portion of the population received the same benefit as the vaccinated portion, with greater than 1 indicating a higher benefit in the vaccinated portion. In simulations with higher vaccine effectiveness, the benefit was greater in the vaccinated compared with the unvaccinated portion of the population than in simulations with lower vaccine effectiveness.

**Table.** Attack Rates With Different Transmission Levels and Different Values of Vaccine Effectiveness

Approximate Rt	AR with no vaccination, mean (SD), %	Vaccine effectiveness, %	AR, mean (SD), %			AR ratio of unvaccinated to vaccinated	Difference in AR, %^a^
			Population	Unvaccinated	Vaccinated		
1.43	26.0 (0.25)	40	13.5 (0.9)	15.9 (1.6)	11.1 (0.9)	1.43	4.8
		45	11.8 (0.9)	14.0 (1.6)	9.7 (0.9)	1.44	4.3
		50	9.7 (1.3)	11.5 (2.3)	7.9 (1.4)	1.45	3.6
		55	8.1 (0.8)	9.7 (1.5)	6.6 (0.8)	1.48	3.2
		60	6.0 (1.0)	7.3 (1.7)	4.8 (1.0)	1.52	2.5
1.81	30.4 (0.23)	40	18.4 (0.5)	22.4 (0.9)	14.6 (0.4)	1.53	7.8
		45	16.9 (0.6)	20.6 (1.1)	13.4 (0.5)	1.54	7.2
		50	15.4 (0.6)	18.9 (1.1)	12.0 (0.5)	1.57	6.9
		55	13.4 (0.8)	16.6 (1.4)	10.4 (0.7)	1.59	6.2
		60	11.4 (1.2)	14.2 (2.2)	8.7 (1.2)	1.63	5.5
1.88	35.0 (0.25)	40	22.1 (0.2)	27.6 (0.3)	16.8 (0.2)	1.64	10.8
		45	21.0 (0.2)	26.3 (0.4)	16.0 (0.2)	1.65	10.4
		50	19.8 (0.3)	24.8 (0.6)	14.9 (0.2)	1.67	10.0
		55	18.3 (0.4)	23.1 (0.9)	13.6 (0.3)	1.69	9.4
		60	16.5 (0.6)	21.0 (1.2)	12.1 (0.5)	1.73	8.8

Abbreviations: AR, attack rate; Rt, reproductive rate.

^a^ The AR in unvaccinated minus AR in vaccinated.

### Levels of Transmission

It has been reported that at very high rates of transmission, the indirect benefit of vaccination may be minimal.^11^ At higher overall influenza case burden, which might be expected in a pandemic situation (51.1% and 85.4% AR with no vaccination, approximate Rt = 2.01 and 3.92, respectively), protection was provided to the entire population and the vaccinated population over a range of values of vaccine effectiveness (eTable 15 in Supplement 1). Indirect protection was lower than for less transmissible strains, with an AR ratio of unvaccinated to vaccinated of 1.88 to 2.54, respectively. When the AR was much higher (93.6% and 96.6% AR, approximate Rt = 4.99 and 5.04, respectively), virtually all of the benefit of vaccination accrued to the vaccinated portion of the population, and the unvaccinated portion was essentially unprotected, even when reduction in AR in the vaccinated group, while less than for lower transmission scenarios, was still substantial (reduction of 52.6% to 61.0% in vaccinated agents, depending on vaccine effectiveness) (eTable 15 in Supplement 1). Indirect benefit essentially disappeared between an approximate Rt of 2.01 and 3.92 (eFigure 9 and eTable 16 in Supplement 1).

## Discussion

In this decision analytical modeling study, we report the averted case burden of modeled influenza vaccination over a range of strain transmissibility, vaccine effectiveness, and vaccine uptake, including the overall averted burden and the averted burden for both the vaccinated and unvaccinated portions of the population. While the direct benefit of vaccination can be estimated from data on cases in vaccinated individuals, the indirect benefit of vaccination, while important, is more difficult to assess.^19^ As has been previously noted, the effects of vaccination can be measured at the population level (change in total cases) in the vaccinated and unvaccinated groups (change in cases in each group) and as a ratio of benefit in the unvaccinated group to benefit in the vaccinated group.^11^ In addition to reporting overall population-level averted burden, we reported the AR ratio of unvaccinated to vaccinated as a measure of the estimated excess benefit to the vaccinated population.

Vaccination surveillance and trial studies have supported the existence of indirect protection of vaccination, particularly in the specific case of vaccination of children, who are drivers of influenza transmission.^3,5,10,20^ A literature review of modeling studies reported the indirect effect of vaccination using a variety of modeling methodologies.^9,10,11,21,22,23,24,25^ Estimates of the magnitude of indirect benefit are mixed, with some studies supporting a larger benefit to unvaccinated than vaccinated populations,^10^ which we did not see in our study.

Most modeling studies that estimated indirect benefit vaccinated only 1 age group, generally children, and estimated the impact on other age groups.^2,3,4,6,9,10,25,26,27,28^ Using a compartmental model and vaccinating all age groups, Arinaminpathy et al^21^ estimated much greater indirect than direct benefits of vaccination. Possibly, the difference from our results may be accounted for by differences in model implementation, specifically vaccine effectiveness and infectious contacts, which only occur in FRED within specific mixing groups (household, school, workplace, and neighborhood), rather than having all infectious agents contact all susceptible agents. Scutt et al^23^ also used a different modeling methodology from ours, which again included universal mixing but reached a similar conclusion to ours when a similar proportion of the population was vaccinated and vaccine effectiveness was in a similar range. Lin et al^11^ also used a compartmental modeling methodology but, again, reached similar conclusions when the direct and indirect benefits were calculated on an individual basis. Due to the highly granular nature of agent-based models, as well as the more organic method of transmission and more realistic mixing patterns of our simulation, our results may be a useful addition to the understanding of both averted burden and direct and indirect benefits of vaccination.

In our results, a substantial proportion of infections was prevented when transmissibility was similar to that encountered in typical influenza seasons, vaccine effectiveness was within the ranges estimated for those seasons, and vaccine uptake was similar to what is typically found in the US. The greatest percent averted burden in the entire population was unsurprisingly found with the combination of highest vaccine effectiveness, lowest level of transmissibility, and highest vaccine uptake, including protection provided to both the vaccinated and unvaccinated portions of the population. Although substantial, the indirect benefit to the unvaccinated population was always less than the direct benefit to the vaccinated population. Indirect benefit in our results was greatest when both virus transmissibility and vaccine effectiveness were low. When transmissibility levels were much higher, as might be expected in a pandemic situation or with a more transmissible pathogen, indirect benefits to the unvaccinated population decreased, and at the highest levels, the indirect benefit was no longer seen.

Our results suggest that a strategy designed to protect the more vulnerable members of the public by allowing lower mortality groups to reach high enough levels of immunity to provide protection via herd immunity when vaccines are unavailable may not be successful for pathogens with high transmission rates. Additionally, since vaccine effectiveness is estimated when a vaccine is used in a population with vaccinated and unvaccinated individuals, the indirect benefit of vaccination may make the estimation of vaccine effectiveness less accurate since unvaccinated individuals may be protected to some extent by decreased levels of transmission overall due to vaccination of a subset of the population.^29,30^

### Strengths and Limitations

While direct and indirect benefits of vaccination have been investigated previously, our agent-based study has several advantages. Our study used an agent-based model, in contrast to the majority of other modeling studies, and so provides information that is complementary to but may differ from other methodologies. Outbreaks are generated by interactions in the simulation population, allowing important facets of the simulation to be produced rather than being imposed. The FRED platform has been used to model numerous infectious disease scenarios, both for influenza and other diseases. In FRED, in addition to estimating total overall reduction in burden, it is possible to identify the direct vs indirect benefits of vaccination in vaccinated and unvaccinated subgroups.

Our study also has some limitations. All models are simplifications of reality; thus, they are useful only in so far as they capture the defining characteristics of the modeled system. Parameterization of models is always difficult but is even more so with influenza since individual influenza seasons are quite variable. To overcome this limitation, we presented scenarios that included ranges of transmissibility, vaccine effectiveness, and vaccine uptake to estimate the variability of results over those parameters. We also performed sensitivity analyses that may be useful in assessing the validity of our results.

## Conclusions

In this decision analytical modeling study, vaccination against influenza revealed considerable benefit in reducing infections in both vaccinated and unvaccinated portions of the population when transmission levels were characteristic of seasonal influenza, underscoring the importance of vaccination in disease prevention and control. However, when the level of transmission was very high, even a highly effective vaccine, while protecting vaccinated individuals, did not protect those who were unvaccinated.

## Notes

Supplement 1. **eMethods. **Overview, Design Concepts, and Details for Framework for Reconstruction Epidemiologic Dynamics Influenza Model
eReferences 1.
**eFigure 1.** Influenza Model State Diagram **eFigure 2.** Simulation Timeline **eTable 1.** Influenza Model Inputs **eFigure 3.** Representative Epidemic Curves for 100 Iterations of Identical Models **eTable 2.** Influenza Burden by Year and Strain Type **eTable 3.** Influenza Model Reproductive Rate (Rt) With Residual Immunity From Prior Infection but No Vaccination **eTable 4.** Comparison of FRED 2010 Allegheny County, PA, Population With US 2020 Census **eTable 5.** Influenza Model Immunity From Prior Year Infection **eTable 6.** Percentage of Agents Vaccinated by Age in Base Scenario and in Decreased and Increased Vaccination Scenarios **eTable 7.** Simulation Parameters Used for Sensitivity Analyses **eFigure 4.** Total Cases With Varied Transmissibility Parameter **eFigure 5.** Total Cases With Varied Percentage of Infections Being Asymptomatic in Different Age Groups **eFigure 6.** Effect on Total Cases of Changes in Length in the Infectious Asymptomatic State Over Different Values for FRED Transmissibility Parameter **eFigure 7.** Impact of Varying the Relative Infectiousness of Asymptomatic Cases **eTable 8.** Percent Difference in Total Cases Between Scenarios With and Without Immunity From Prior Infection **eFigure 8.** Cumulative Vaccination Uptake as Reported by FluVaxView **eTable 9.** Sensitivity Analysis of Timing of Vaccine Distribution **eTable 10.** Characteristics of Additional Simulation Counties **eTable 11.** Relationship of FRED Transmissibility Parameter to Attack Rate (AR) in Counties With Different Demographics in the Influenza Model With Only Difference Being Either Transmissibility Parameter or County Structure **eTable 12.** Ratio of Attack Rate (AR) in Unvaccinated to AR in Vaccinated in Counties With Different Demographics Over Varied Base ARs With No Vaccination and Varied Values of Vaccine Efficacy, Using Base Influenza Model **eTable 13.** Percent Reduction in Influenza Burden Due to Vaccination, With Varied Transmissibility of the Strain (~Rt) and Effectiveness of the Vaccine **eTable 14.** Percentage of Total Cases Averted by Vaccination With Varied Vaccine Uptake **eTable 15.** Direct and Indirect Benefits of Vaccination in High Transmission Scenarios **eFigure 9.** Decrease in Indirect Benefit of Vaccination as ~Rt Increases From 2.01 to 3.92 **eTable 16.** Relationship of ~Rt to Change in Amount of Direct and Indirect Benefit of Vaccination
eReferences 2.

Supplement 2.
Data Sharing Statement
