# Supplementary material for: Estimated Burden of Influenza and Direct and Indirect Benefits of Influenza Vaccination
Source: JAMA Netw Open. 2025 Jul 16;8(7):e2521324. doi: 10.1001/jamanetworkopen.2025.21324 (PMC12268489; doi:10.1001/jamanetworkopen.2025.21324)
Supplement: Supplement 1. — eMethods. Overview, Design Concepts, and Details for Framework for Reconstruction Epidemiologic Dynamics Influenza Model eReferences 1. eFigure 1. Influenza Model State Diagram eFigure 2. Simulation Timeline eTable 1. Influenza Model Inputs eFigure 3. Representative Epidemic Curves for 100 Iterations of Identical Models eTable 2. Influenza Burden by Year and Strain Type eTable 3. Influenza Model Reproductive Rate (Rt) With Residual Immunity From Prior Infection but No Vaccination eTable 4. Comparison of FRED 2010 Allegheny County, PA, Population With US 2020 Census eTable 5. Influenza Model Immunity From Prior Year Infection eTable 6. Percentage of Agents Vaccinated by Age in Base Scenario and in Decreased and Increased Vaccination Scenarios eTable 7. Simulation Parameters Used for Sensitivity Analyses eFigure 4. Total Cases With Varied Transmissibility Parameter eFigure 5. Total Cases With Varied Percentage of Infections Being Asymptomatic in Different Age Groups eFigure 6. Effect on Total Cases of Changes in Length in the Infectious Asymptomatic State Over Different Values for FRED Transmissibility Parameter eFigure 7. Impact of Varying the Relative Infectiousness of Asymptomatic Cases eTable 8. Percent Difference in Total Cases Between Scenarios With and Without Immunity From Prior Infection eFigure 8. Cumulative Vaccination Uptake as Reported by FluVaxView eTable 9. Sensitivity Analysis of Timing of Vaccine Distribution eTable 10. Characteristics of Additional Simulation Counties eTable 11. Relationship of FRED Transmissibility Parameter to Attack Rate (AR) in Counties With Different Demographics in the Influenza Model With Only Difference Being Either Transmissibility Parameter or County Structure eTable 12. Ratio of Attack Rate (AR) in Unvaccinated to AR in Vaccinated in Counties With Different Demographics Over Varied Base ARs With No Vaccination and Varied Values of Vaccine Efficacy, Using Base Influenza Model eTable 13. Percent Reduction in Influenza Burden [file jamanetwopen-e2521324-s001.pdf]

Supplemental Online Content

Krauland MG, Mandell A, Roberts MS. Averted burden of influenza and direct and indirect benefits of vaccination. *JAMA Netw Open*. 2025;8(7):e2521324. doi:10.1001/jamanetworkopen.2025.21324

eMethods. Overview, Design Concepts, and Details for Framework for Reconstruction Epidemiologic Dynamics Influenza Model

eReferences 1

eFigure 1. Influenza Model State Diagram

eFigure 2. Simulation Timeline

eTable 1. Influenza Model Inputs

eFigure 3. Representative Epidemic Curves for 100 Iterations of Identical Models

eTable 2. Influenza Burden by Year and Strain Type

eTable 3. Influenza Model Reproductive Rate (Rt) With Residual Immunity From Prior Infection but No Vaccination

eTable 4. Comparison of FRED 2010 Allegheny County, PA, Population With US 2020 Census

eTable 5. Influenza Model Immunity From Prior Year Infection

eTable 6. Percentage of Agents Vaccinated by Age in Base Scenario and in Decreased and Increased Vaccination Scenarios

eTable 7. Simulation Parameters Used for Sensitivity Analyses

eFigure 4. Total Cases With Varied Transmissibility Parameter

eFigure 5. Total Cases With Varied Percentage of Infections Being Asymptomatic in Different Age Groups

eFigure 6. Effect on Total Cases of Changes in Length in the Infectious Asymptomatic State Over Different Values for FRED Transmissibility Parameter

eFigure 7. Impact of Varying the Relative Infectiousness of Asymptomatic Cases

eTable 8. Percent Difference in Total Cases Between Scenarios With and Without Immunity From Prior Infection

eFigure 8. Cumulative Vaccination Uptake as Reported by FluVaxView

eTable 9. Sensitivity Analysis of Timing of Vaccine Distribution

eTable 10. Characteristics of Additional Simulation Counties

eTable 11. Relationship of FRED Transmissibility Parameter to Attack Rate (AR) in Counties With Different Demographics in the Influenza Model With Only Difference Being Either Transmissibility Parameter or County Structure

© 2025 Krauland MG et al. *JAMA Network Open*.

eTable 12. Ratio of Attack Rate (AR) in Unvaccinated to AR in Vaccinated in Counties With Different Demographics Over Varied Base ARs With No Vaccination and Varied Values of Vaccine Efficacy, Using Base Influenza Model

eTable 13. Percent Reduction in Influenza Burden Due to Vaccination, With Varied Transmissibility of the Strain (~Rt) and Effectiveness of the Vaccine

eTable 14. Percentage of Total Cases Averted by Vaccination With Varied Vaccine Uptake

eTable 15. Direct and Indirect Benefits of Vaccination in High Transmission Scenarios

eFigure 9. Decrease in Indirect Benefit of Vaccination as ~Rt Increases From 2.01 to 3.92

eTable 16. Relationship of ~Rt to Change in Amount of Direct and Indirect Benefit of Vaccination

eReferences 2

This supplemental material has been provided by the authors to give readers additional information about their work.

© 2025 Krauland MG et al. *JAMA Network Open*.

eMethods. Overview, Design Concepts and Details for Framework for Reconstruction Epidemiologic Dynamics Influenza Model

1. Purpose and patterns

The purpose of this model is to estimate the averted burden and both direct and indirect benefit of influenza vaccination over a range of virus transmissibilities and values of vaccine effectiveness. This set of models employs a range of values for these parameters to reflect the variability of transmissibility of seasonal and pandemic influenza and the variability in possible levels of vaccine effectiveness.

Two patterns were used to evaluate this set of models. First, we calibrated to a reasonable estimate of a seasonal influenza outbreak. Since such outbreaks are highly variable, this demonstrates only that the model can be calibrated to reasonably reproduce a seasonal influenza outbreak. The second pattern evaluates whether the results are logical over a range of parameters and that they are informative in terms of describing the benefit of vaccination over that wider range.

2. Entities, state variables, and scales

The main entities in the model are agents, representing people. In FRED populations, agents represent people whose demographics and household locations are obtained from the census through iterative proportion fitting, which results in realistic but deidentified populations. Agents have school and workplace assignments which are derived from appropriate data sources. Additionally, FRED models have meta-agents who in this model start disease outbreaks by infecting selected individuals. Meta-agents have no further or other roles in this model and have no state variables.

Since the model deals with influenza in humans. those entities are necessary and other entities are not included because they do not play a role in influenza transmission.

Entity State Variables

variable	type / units	Description
gender	Binary, static	-
age	Continuous, dynamic	-
race	static	-
income	static	-
susceptibility to infection	Continuous, dynamic, range 0-1	Generated by combination of prior immunity, vaccination and infection

The model represents time as 24 hour time steps. Each scenario ran for 289 day steps.

Spatial resolution for agents is granular within the location. For this model most scenarios take place in Allegheny County although some scenarios use other US counties to determine if results were consistent in other locations. Households have exact latitude and longitude, as do schools and workplaces. Household locations are not exact matches to real houses but are realistically placed based on real household data. Schools are located at actual school addresses. Workplaces are situated similarly to households; that is, realistically located but not in the location of a real business. FRED agents also interact in neighborhoods. The simulation geography is divided into $1km^2$ neighborhood areas; each household resides in a neighborhood and its inhabitants interact with other agents who also reside in the neighborhood. Neighborhoods are intended to include ad-hoc interactions, both business and social.

Allegheny County, Pa, USA, is a county that includes urban and suburban areas. The population size is 1,218,695. The demographic makeup of the county is proportionally similar to that of the 2020 US census population.

Other dimensions are not represented.

The specific spatial scale was chosen to provide a large enough population to demonstrate the dynamics of seasonal influenza while being computationally tractable.

The temporal scale was chosen to encompass a typical respiratory infection season (August 15 to May 31).

3. Process overview and scheduling

The model encompasses one respiratory disease season. The processes include distribution of immunity from prior year infection, vaccination and infection.

FRED keeps track of each agent's current state in each sub model. For each state, rules control how agents change state or update variables during a FRED simulation. There are three categories of rules in FRED: Action Rules, Wait Rules, and Transition Rules. Action Rules control how the agent's own variables change when the agent enters a state. Wait Rules control how long an agent stays in a given state. Transition Rules control how an agent moves from one state to another.

In this model, each state has a wait time, some of which are variable. During each 24 hour day of the simulation, each agent will accomplish all the state transitions that are appropriate for their current states and for how long they have been in those states. Prior immunity is distributed at initialization and contains no further state changes after that. Agents either receive a reduction is susceptibility to infection or they do not. Vaccination is scheduled early in the simulation time course to roughly mimic the uptake schedule reported by the Centers for Disease Control and Prevention. Cases are imported in mid-October so that a seasonal outbreak will have peak timing and epidemic curve of infections that is similar to that of influenza. Once cases are imported, susceptible agents who share mixing groups (household, school, workplace or neighborhood) can be infected by contact with infectious agents. Infection is determined for each infectious contact

© 2025 Krauland MG et al. *JAMA Network Open*.

by considering the infectivity of the infector (full for symptomatic infected agents or 50% for presymptomatic or asymptomatic infected agents). Number of infectious contacts per infected agent also depends on contact rates in the specific mixing group and on the length of time the agents spend in that mixing group during the daily time step.

Agents accomplish the evaluation of state and state changes in order; that is, first agent to last agent and that order is the same for each timestep.

The model includes the following processes:

1. Prior immunity which represents prior year infection and is distributed when the simulation starts
    a. Agents are chosen to have reduced susceptibility to infections at age-appropriate rates based on number of infections reported by the CDC in the 2019-2020 flu season
    b. This is accomplished in a single time step upon initialization.
    c. Agents either receive decreased susceptibility or do not
    d. Prior immunity wanes at a rate of 3% per month for agents not vaccinated or infected
    e. If vaccinated or infected, waning will be controlled by those sub models, not by the prior immunity model
2. Vaccination
    a. Begins on September 20 in the simulation
    b. Agents are chosen randomly to vaccinate at age-group specific rates derived from CDC reports
    c. Agents who are not vaccinated go to a non-vaccinated state
    d. Vaccinated agents go to a wait state
    e. Agents in the Vaccinated state progress to the vaccination state with a uniform distribution over 45 days
    f. 14 days after vaccination, agent's susceptibility to infection is reduced by an amount equal to the modeled value for vaccine effectiveness
    g. Agents who have reduced susceptibility due to prior immunity receive an addition reduction in susceptibility due to vaccination at a reduced rate described by: current susceptibility - (current susceptibility * vaccine effectiveness)
    h. Every 30 days a vaccinated agent's susceptibility increases by 7% to represent waning of immunity
3. Influenza
    a. Begins with the importation of 50 initial cases on October 15 of the simulation
    b. Prior work in this model indicated that 50 cases would reliably produce reasonably sized outbreaks

c. October 15 was chosen to produce epidemic curves and peak transmission that are realistic for seasonal influenza
d. Seasonal influenza is highly variable in timing so we chose to match timing to an average season
e. In each day step susceptible agents may be infected if there is an infectious agent in one or more of their mixing locations.

4. StayHome

   a. Symptomatic agents isolate at home with a probability of 50% until recovered
   b. The influenza condition sends symptomatic infected agents to the StayHome sub model
   c. Influenza also moves agents from the isolating state back to normal when the agent transitions to the recovered state

Actions are scheduled to represent the timing in reality. Prior immunity would be pre-existing so is distributed immediately upon simulation initialization. Vaccination is designed to mimic the actual time course of both immunization and the lag to development of immunity. Day steps allow interactions to spread infection. The sequence of state transitions in the influenza infection are designed to mimic the natural history of infection.

5. Design concepts

   a. Basic principles

This model uses individual agent interactions to transmit infection in modeled mixing groups. These mixing groups have contact rates that influence the likelihood of transmission in their specific location. This methodology allows a natural and organic transmission of disease. The questions posed to this model are what is the burden of infection that is prevented by vaccination and what is the direct and the indirect benefit that accrues to the vaccinated and unvaccinated portions of the population, respectively. Influenza has highly variable seasonal burden and has the potential for very high disease burden in the pandemic setting. For this reason, the model uses a range of both virus transmissibilities and vaccine effectiveness levels to explore a range of transmission scenarios rather than focusing on 1 or 2 commonly encountered seasonal scenarios.

Simulations include a main infection model with additional sub models to represent additional aspects of viral transmission.

The model provides insights into the basic principles that govern the interplay of vaccination and infection.

The behavior aspect of this model is limited. Agents only behavior, other than interactions in mixing groups, is to isolate at home at a rate of 50% when symptomatic, a rate derived from the literature [1]. The decision to isolate is purely probabilistic.

   b. Emergence

© 2025 Krauland MG et al. *JAMA Network Open*.

The key outcome is total infections, which emerges through interactions of infected and susceptible agents in the mixing groups of households, school, workplaces and neighborhoods.

c. Adaptation

The only adaptive behavior in this model is isolating at home when symptomatic, which takes place for 40% of symptomatic agents. This value was derived from data reported by the CDC [1].

d. Objectives

Agents do not engage in behaviors in which they assess any objective measures so objectives are not implemented in this model.

e. Learning

Learning is not implemented in this model, as there is no adaptive behavior.

f. Prediction

Prediction is not implemented in this model.

g. Sensing

Sensing is not implemented in this model.

h. Interaction

In this model, transmission of an infectious condition is accomplished through agent interactions in location specific mixing groups (households, schools, workplaces and neighborhoods). Each location has hours during which interactions can take place and has contact rates which were obtained through calibration to produce realistic age-group specific rates of infection.

i. Stochasticity

Stochasticity is produced in the model through random choice of initial infected agents and by the use of random probability draws to determine transmission of infection. A transmission probability is calculated based on the place-specific contact rate, the transmissibility of the condition and the number of hours in the interaction time block. The product (transmission probability * agent susceptibility) is compared to a random draw to decide if an interaction transmits infection. Random probability draws add stochasticity in 2 ways: by causing agents with different demographics and mixing group membership to be selected for initial infection and by influencing the choice of different agents to become infected through transmission.

j. Collectives

Collectives in this model are represented by the FRED mixing groups: households, schools, workplaces and neighborhoods. These collectives act as the basis for agent interactions that

© 2025 Krauland MG et al. *JAMA Network Open*.

accomplish infection transmission. Each of these collectives has its own characteristic time frame and contact rate.

k. Observation

The simulations produce results specific to each model and sub model. The results are structured as comma separated value files that include incidence, prevalence and totals for each state in each included model/sub model for each day of the simulation. Results include individual runs in the simulation as well as quantiles, mean and standard deviation from the multiple runs.

6. Initialization

Upon initialization agents are read in from a specified location specific population file. The base location level is a US county; however, aggregations of counties or sub-county level populations may also be used. Agents have demographic information (age, gender and race) and are part of households. They are assigned to schools and workplaces as appropriate for their ages. Each block group level geographic area has household number, composition and income that is statistically similar to the census data. Households have locations which mimic real household locations based on geographic household location data but the system does not use actually household address. The result is that households are not located in unrealistic locations such as bodies of water or freeways. Agent data is derived from the census and is associated with the agent so is an input and not variable between model runs. Methodology for population creation has been documented extensively elsewhere [2-5]. Initialization is always the same for the same geographic location but different populations are used for different locations.

Agents are assigned to 1 km^2 neighborhood mixing groups. Neighborhoods are set up at run time.

7. Input data

The model does not use input data to represent time-varying processes.

8. Submodels

a. Influenza is modeled as follows:

i. Initial cases are imported by a random choice of susceptible agents
ii. FRED uses the place-specific contact rate, the transmissibility of the condition and the number of hours in the contact time block to decide how many infection attempts to make for each infectious agent
iii. FRED makes a list of infectious agents and makes infection attempts in each of their mixing groups
iv. FRED uses age-bias of the infector and infectee to calculate the transmission probability.
v. Transmission probability * agent susceptibility is compared to a random draw to decide whether an agent is infected

vi. The model includes a seasonal forcing mechanism which moderates the FRED transmissibility parameter
vii. Transmissibility is modified by a multiplier calculated by the equation: seasonal_reduction*(1 - 0.5*(1+cos(2*3.14159*days_from_peak/365))) where the maximum seasonal reduction is 0.5 and peak day of transmission in the year is day 355 [6]
viii. Susceptible agents who are infected transition to the Exposed state, which represents latent infection. Agents are not infectious in this state
ix. Agents wait for a time drawn from a lognormal(1.9,1.23) distribution
x. Agents transition to the asymptomatic state at a rate of 25% and remain there for a wait period drawn from a lognormal(4.0, 1.5) distribution
xi. Asymptomatic agents are 50% as infectious as Symptomatic agents
xii. Asymptomatic agents all transition to Recovered
xiii. Remaining agents transition to the Presymptomatic infectious state, in which they are 50% as infectious as Symptomatic agents where they remain for 1 day
xiv. Presymptomatic agents transition to the Symptomatic state where they are fully infectious
xv. Symptomatic agents transition a wait period drawn from a lognormal(5.0,1.5) distribution
xvi. Some Symptomatic agents transition to Hospitalized
xvii. Remaining Symptomatic agents transition to Recovered
xviii. Hospitalized agents transition to Recovered or Died after a 0 length wait period because this state is used only for counting
xix. Died state is also 0 wait time and only used for counting
xx. Agents who die are removed from the population
xxi. While there is no formal return to the susceptible state, recovered agents may be reinfected depending on their susceptibility due to waning of immunity.

b. Prior immunity – simulates effect of prior year infection
    i. Agents are randomly chosen for prior immunity at age-group specific rates that are similar to the proportion of people who are infected each year [7]
    ii. Agents with prior immunity have their susceptibility reduced by 50%, a value derived from serology and expert opinion [8]
    iii. Agents who do not receive prior immunity have no changes in susceptibility

c. Vaccination
    i. Agents are chosen for vaccination at age-group specific rates based on vaccine uptake reported by the CDC for the 2019-2020 season (https://www.cdc.gov/flu/fluvaxview/coverage-1920estimates.htm)

© 2025 Krauland MG et al. *JAMA Network Open*.

ii. Agents chosen for vaccination proceed to a wait state drawn from a uniform distribution. (1,45)
iii. After waiting, agents proceed to vaccination
iv. Agents receive a reduction in susceptibility to infection equal to specified vaccine effectiveness
v. Immunity wanes every 30 days at a rate of 7%
vi. Agents with prior immunity who are also vaccinated receive a boost in immunity proportional to how much protection they have at the time of vaccination
vii. Agents with prior immunity who are also vaccinated will have immunity wane at 7% until they reach the level of immunity they had before vaccination
viii. Further waning is at 3% per month

d. Isolate at home (StayHome)
    i. Symptomatic agents isolate at home at a rate of 50% [1]
    ii. Isolated agents interact only in their household and are absent from other mixing groups
    iii. Agents return to normal interactions when they transition to the R state
    iv. Initiation of isolation and return to normal are controlled by the influenza model

The prior immunity and vaccination sub models were tested in isolation. These are probability based models that do not require the infection model to produce results. Testing ensures that the proper number of agents are given the designated difference in susceptibility to infection. These values were tested for stability over 100 runs and display only slight variations. The isolate at home model was tested by combining with a simple infection model. The number and timing of agents isolated was compared to the number of infections to ensure that the appropriate number of agents isolated.

eReferences 1

1. Ahmed F, Kim S, Nowalk MP, et al. Paid Leave and Access to Telework as Work Attendance Determinants during Acute Respiratory Illness, United States, 2017-2018. Emerg Infect Dis. 2020;26(1):26-33.
2. Cajka JC, Cooley, P. C., & Wheaton, W. D. Attribute assignment to a synthetic population in support of agent-based disease modeling. RTI Press;2010.
3. Chasteen BM, Wheaton, W. D., Cooley, P. C., Ganapathi, L., & Wagener, D. K. Including the group quarters population in the US synthesized population database. RTI Press;2011. RTI Press Methods Report No. MR-0020-1102
4. Chrest DP, & Wheaton, W. D. . Using geographic information systems to define and map commuting patterns as inputs to agent-based models. RTI Press;2009.
5. Wheaton WD, Cajka, J. C., Chasteen, B. M., Wagener, D. K., Cooley, P. C., Ganapathi, L., Roberts, D. J., & Allpress, J. L. . Synthesized population databases: A US geospatial database for agent-based models. RTI Press;2009.
6. Kissler SM, Tedijanto C, Goldstein E, Grad YH, Lipsitch M. Projecting the transmission dynamics of SARS-CoV-2 through the postpandemic period. *Science.* 2020;368(6493):860-868.
7. Centers for Disease Control and Prevention. Estimated flu disease burden, by age group — United States, 2019-2020 flu season. https://www.cdc.gov/flu/about/burden/2019-2020.html. Accessed July 28, 2022.
8. Tsang TK, Perera R, Fang VJ, et al. Reconstructing antibody dynamics to estimate the risk of influenza virus infection. *Nat Commun.* 2022;13(1):1557.

© 2025 Krauland MG et al. *JAMA Network Open*.

Supplementary Materials

1. Framework for Reconstructing Epidemiologic Dynamics (FRED) Details
2. Influenza Model Description
3. Influenza burden
4. FRED Reproductive Rate
5. Residual Immunity from Prior Infection
6. Vaccination
7. Sensitivity Analysis
8. Populations with Different Demographics
9. Averted Burden Additional Results

1. Framework for Reconstructing Epidemiologic Dynamics (FRED) Details

FRED is an agent-based modeling platform that uses realistic census-based populations that are statistically equivalent to real populations in gender, age, household size and composition and household location. FRED populations were developed using data from the 2010 census as well as the American Community Survey, with additional information on school and workplace size and location [2-5]. Agents interact in their household, school and workplace locations as well as in a 1-square kilometer partition of the simulation location which includes their household location, which FRED refers to as a neighborhood.

Each FRED model is a collection of states, state transition probabilities and wait times that describe a disease process. Simulations can contain a collection of models that may or may not interact. Individual models can affect state changes in other models. Agents are in exactly one state in each included model during each day of a simulation.

2. Influenza Model Description

This study used an extended SEIR model influenza model (eFigure 1). The model included a pre-symptomatic period of 1 day when agents are 50% as infectious as agents in the Symptomatic state. An additional state represents asymptomatic infection, also 50% at infectious as Symptomatic agents. Infected agents transition to the Pre-symptomatic state with a probability of 75% followed by transitioning to the Symptomatic state; 25% of infected agents transition to the asymptomatic state. Symptomatic agents may transition to the Hospitalized state; some agents in this state die. Neither hospitalization nor death were analyzed outcomes in this study. While there is no formal return to the susceptible state, agents may be reinfected depending on their susceptibility due to waning of immunity.

All simulations start on August 15, a date chosen to give time for vaccination to be completed before the start of the influenza season. Vaccination begins on September 20. An initial import of 50 cases to random agents in the population occurs on October 15 of the simulation to begin the influenza season. eFigure 2 shows a timeline of the simulation. 100 simulations were run for each parameter set. Model inputs are described in eTable 1.

The influenza model includes a seasonality component that moderates transmissibility based on the days from the approximate date of the winter solstice, with a maximum transmissibility on day 355 of the simulation year and a minimum at the approximate summer solstice (183 days from the winter solstice) [6].

The asymptomatically infectious state is an important part of the overall influenza model. While it is generally accepted that some influenza cases are asymptomatic, reported estimates of the rate of asymptomatic disease are widely varied [9,10]. As a conservative estimate, the model transitions infected agents to the asymptomatic state at a rate of 25% and asymptomatic agents are infectious at 50% the level of symptomatic cases. This value is supported by some data but is not universally accepted. One study reported that 27% of secondary influenza infections were

© 2025 Krauland MG et al. *JAMA Network Open*.

acquired from asymptomatic cases [11]. Our method provides a reasonable approximation for this rate. Results of limited sensitivity analysis of characteristics related to the asymptomatic state are located in Section 7.

FRED software is available at: https://github.com/PublicHealthDynamicsLab/FRED. Example code for this project is available at:
https://github.com/PublicHealthDynamicsLab/Models/tree/f5364573803258f4cb3f6fa4cbd2c565a6a4afb4/averted_burden.

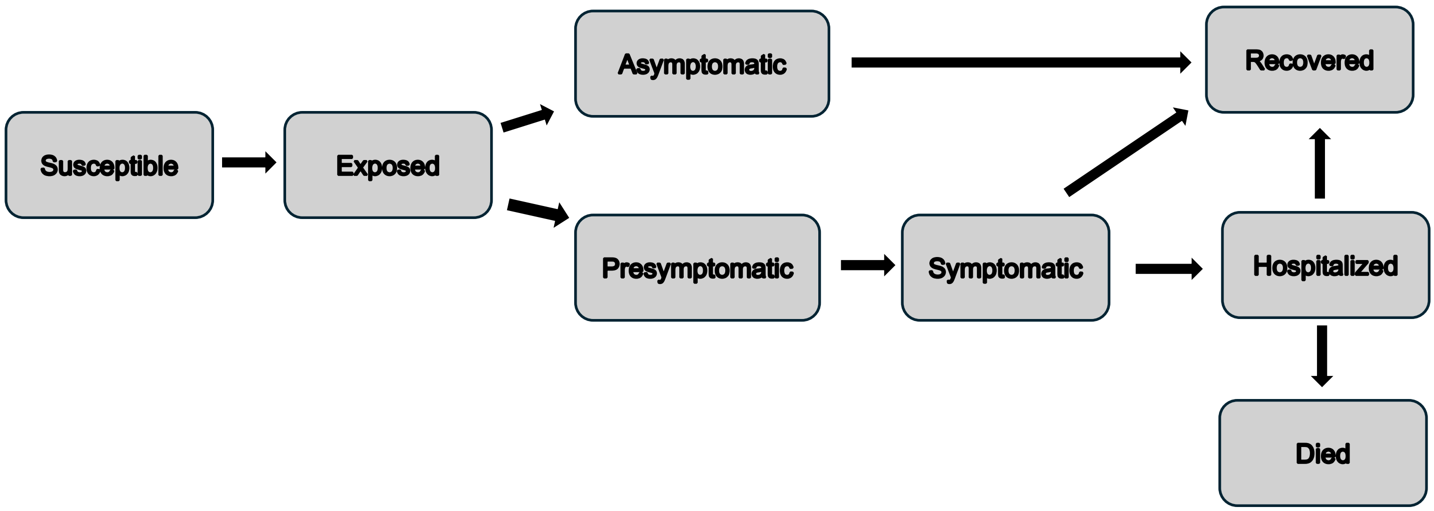

eFigure 1. Influenza Model State Diagram

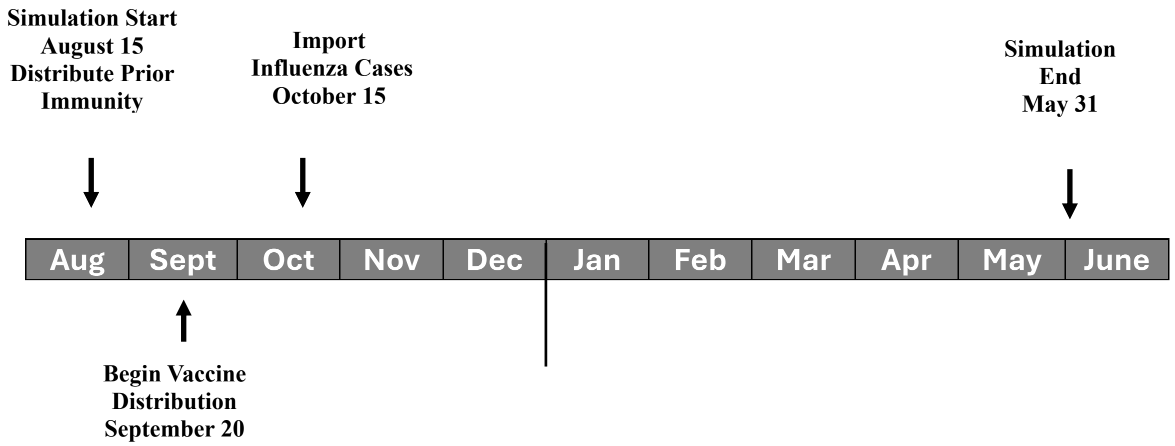

eFigure 2. Simulation Timeline

© 2025 Krauland MG et al. *JAMA Network Open*.

eTable 1. Influenza Model Inputs

Inputs	Details	References
Population	1,218,695 agents derived from Allegheny County 2010 census population	[2-5]
**Susceptibility-related parameters**		
Susceptibility to infection	1	
Susceptibility with prior immunity	Normal distribution: $\mu = 0.50$, $\sigma = 0.10$	[8]
Rate by age for assigning prior immunity by age in years	0-1, 0%; 2, 12.54%; 3-4, 25.08%; 5-17, 18.48%; 18-49, 13.32%; 50-64, 16.08%; ≥65, 4.32%.	[7]
Waning of immunity from infection	3% monthly	[8]
Vaccine Effectiveness (VE)	Main simulations: 40% to 60% by increments of 5%<br>Additional simulations: 30% to 70 % by 5%	[12]
Waning of Vaccine Efficacy	7% monthly	[13,14]
**Influenza state durations**		
Latent infected (E) in days	Drawn from a lognormal distribution: $\mu = 1.9$, $\sigma = 1.23$	[15]
Pre-symptomatic (Ps) in days	Duration one day	[16]
Symptomatic infectious (Is) in days	Drawn from a lognormal distribution: $\mu = 5$, $\sigma = 1.5$	[10]
Asymptomatic infectious (Ia) in days	Drawn from a lognormal distribution: $\mu = 4$, $\sigma = 1.5$	[10]
**Simulation Parameters**		
Simulation Period	August 15 to May 31	
Simulations Per Scenario	100	

As a validation of calibration for our model, we compared our results to the estimated influenza burden for 2022-23 as reported by the Centers for Disease Control and Prevention (CDC). The estimated number of symptomatic influenza cases for that season was 31,914,978 (95% uncertainty interval: 26,687,734 to 51,822,729) (https://www.cdc.gov/flu-burden/php/data-vis/2022-2023.html). The estimated vaccine efficacy for that season is reported as ranging from 23 to 68%, varying by source of data and age group (https://www.cdc.gov/flu-vaccines-work/php/effectiveness-studies/2022-2023.html). We did not use age-specific vaccine efficacy, instead applying a single rate to all vaccinated agents. Scaling the national case load to Allegheny County gave a value of 117,347 symptomatic cases. When adjusted for 25% asymptomatic cases, the estimate was 146,684 total cases. This was in good agreement with our results for low (~R_t 1.43) and medium transmission (~R_t 1.81) with vaccine effectiveness of 40 or 60% (total cases 164,370, standard deviation 14,943 for ~R_t 1.43 and 40% vaccine efficacy; total cases 143,593, standard deviation 15,255 for ~R_t 1.43 and 45% vaccine efficacy; total cases 163,610, standard deviation 12,423 for ~R_t 1.81 and 55% vaccine efficacy; total cases 138,762, standard deviation 20,104 for ~R_t 1.81 and 60% vaccine efficacy). Representative epidemic curves for several values of ~R_t are shown in eFigure 3. Epidemic curves were generated for listed ~R_t with vaccination at base levels and VE of 40%. Simulations are more variable at lower ~R_t.

© 2025 Krauland MG et al. *JAMA Network Open*.

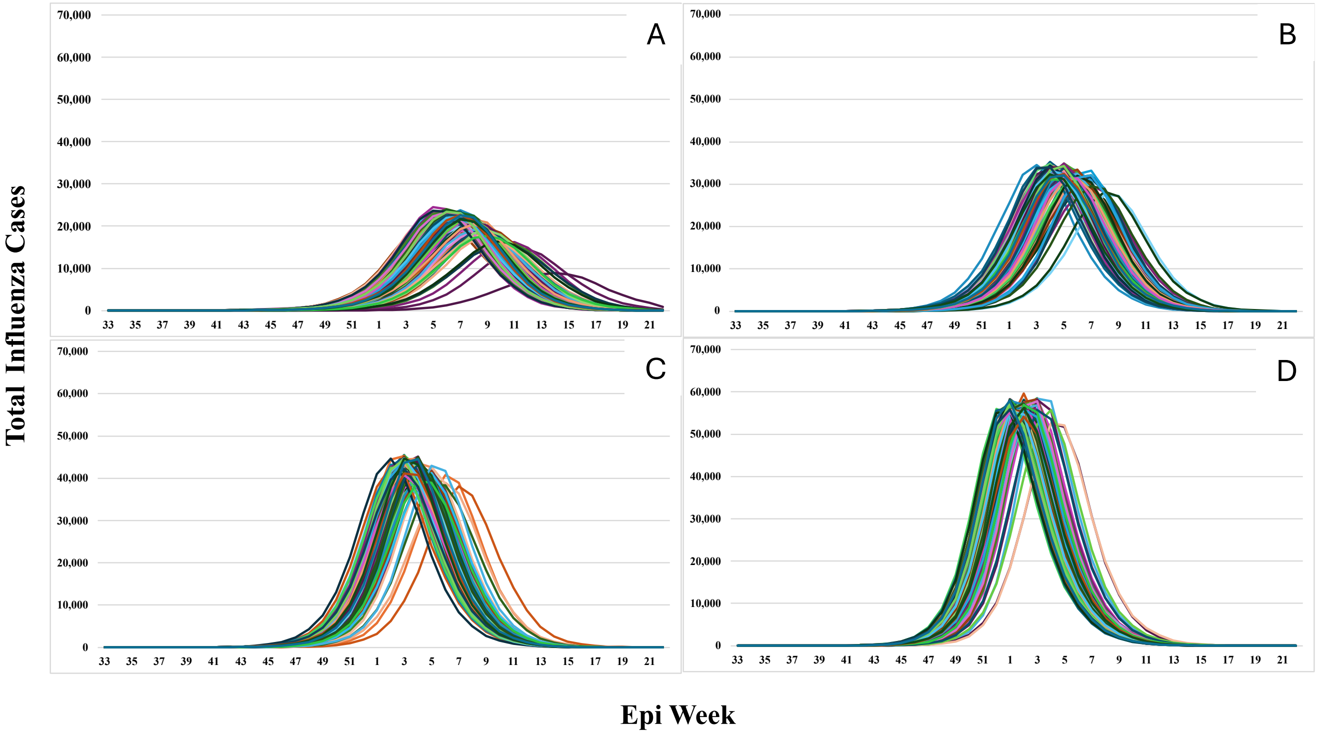

eFigure 3. Representative Epidemic Curves for 100 Iterations of Identical Models for increasing levels of ~R_t. A: ~ R_t = 1.33; B: ~ R_t = 1.43; C: ~ R_t = 1.83; D: ~ R_t = 1.86.

3. Influenza Burden

Influenza burden varies widely by year and by predominant type. Influenza burden typically includes a mixture of types, although usually one type predominates. Over the 14-season period from 2010-11 to 2023-24, 7 seasons had H3N2 as the predominant type A strain, 4 had H1N1 as the predominate type A strain and 2 seasons were mixed H1N1 and H3N2 (eTable 2) [17]. Season 2020-21 did not have a seasonal outbreak due to COVID-19 interventions. The 2019-20 season also had a larger than usual circulation of type B strains. Even within a subtype, the seasonal case burden can vary widely, with H3N2 having both the lowest and highest estimated burdens during that time period (9.3 million in 2011-12 and 41.0 million in 2017-18). This makes identifying a representative season challenging. We therefore report results obtained for a range of values of the FRED transmissibility parameter, to mimic the range of case burden seen in the US.

© 2025 Krauland MG et al. *JAMA Network Open*.

eTable 2. Influenza Burden by Year and Strain Type

Year	Predominant Type	Symptomatic cases[1]
2010-11	Mixed	21.2 (19.9-24.9)
2011-12	H3N2	9.3 (8.7, 12.0)
2012-13	H3N2	33.7 (31.7, 38.0)
2013-14	H1N1	29.7 (27.9, 32.7)
2014-15	H3N2	30.1 (28.6, 32.7)
2015-16	H1N1	23.5 (19.9, 32.5)
2016-17	H3N2	29.2 (24.7, 44.9)
2017-18	H3N2	41.0 (35.5, 53.3)
2018-19	Mixed (H1N1 more common)	28.9 (24.9, 40.5)
2019-20	H1N1 and B	35.9 (28.7, 71.2)
2020-21		No seasonal outbreak
2021-22	H3N2	9.3 (7.7, 15.5)
2022-23	H3N2	31.9 (26.6-51.8)
2023-24[2]	H1N1	35 – 65[2]

[1]In millions, 95% confidence interval in parenthesis
[2]Preliminary estimate, no point estimate available

4. FRED Reproductive Rate

FRED does not impose a reproductive rate; rather the reproductive rate in a simulation is produced as a combination of a number of factors. The reproductive rate of an outbreak is a function of the transmissibility of the pathogen, the characteristics of the disease process (length of latent and infectious periods, presence of asymptomatic transmission, etc) and the characteristics of the population used for simulation (demographics, personal characteristics, interaction patterns and contact rates). This has been previously described [18-20]. Both the reproductive rate and the closely related force of infection combine these factors and so are generated in the simulation rather than being inputs. FRED reports a reproductive rate for each timestep but not a force of infection.

FRED agents represent individuals in the population. FRED populations were generated by a process based on iterative proportional fitting of population characteristics to census data at the block group level [2-5,21]. FRED agents have mixing groups that include households, neighborhoods (1 km^2 areas that include the household location) and schools and workplaces derived from location-specific data. Mixing within these locations depending on contact rates determined by literature and calibration contribute to the production of reproductive rates [19].

Infectious disease models in FRED use a transmissibility parameter to capture the inherent transmissibility of a pathogen. In FRED this parameter is chosen to produce appropriate transmission rates in conjunction with the other relevant parameters and can be varied to produce different case burden within the same model (eTable 3). When all parameters other than transmissibility remain constant, changes to results will be caused by changes in the transmissibility parameter. Scenarios described here were designated by the R_t of a simulation with no vaccination for convenience in comparing them.

© 2025 Krauland MG et al. *JAMA Network Open*.

eTable 3. Influenza Model Reproductive Rate (R_t) With Residual Immunity From Prior Infection but No Vaccination

FRED transmissibility parameter	0.6	0.65	0.7	0.75	0.8	0.85
R_t with residual immunity	1.33 (0.47)[1]	1.43 (0.49)	1.81 (0.39)	1.88 (0.32)	1.96 (0.24)	1.98 (0.14)
Attack Rate in Allegheny County	21.16 (0.89)[1]	26.02 (0.25)	30.39 (0.23)	35.03 (0.25)	40.11 (0.54)	45.49 (0.67)

[1] Standard deviation

The majority of simulations described here used the FRED 2010 Allegheny County population (1,218,695 agents). The FRED Allegheny County population roughly mirrors the age-group makeup of the US 2020 census population (eTable 4) and contains a mix of urban and suburban areas [2-5,21].

eTable 4. Comparison of FRED 2010 Allegheny County, PA, Population With US 2020 Census

Age group	Percent in age group in US[1]	Number in age group in US[2]	Number in age group scaled to PA[3]	Percent in age group in FRED Allegheny County population	Number in age group in FRED Allegheny County population
0-4	4.39%	14,550,623	53,501	5.17%	63,016
5 to 17	16.23%	53,794,218	197,794	15.30%	186,501
18-49	41.91%	138,910,394	510,755	41.49%	505,676
50-64	18.68%	61,914,726	227,652	21.05%	256,540
65 and over	17.70%	58,666,523	215,709	16.98%	206,962

[1] From https://www.census.gov/popclock/data_tables.php?component=pyramid
[2] Based on US total population size of 331,449,281
[3] Scaled to FRED Allegheny County population size of 1,218,695

5. Residual Immunity from Prior Infection

CDC reported rates of infection from the 2019-20 season (https://www.cdc.gov/flu/about/burden/2019-2020.html) were used to set pre-existing immunity using a simple 3 state model (states: Start, Set, Not) at simulation start. Agents transitioning to the Set state are given a 50% reduction in susceptibility to influenza infection at rates by age-group as in eTable 5. Set and Not are absorbing states and agents remain in those states in the prior immunity model for the duration of the simulation. Agents were randomly chosen for the application of prior immunity. Different agents would be chosen randomly from the pool of agents in each age group in each run, such that different agents would receive immunity in each simulation in the set. The number of agents who received prior immunity matched the stated probabilities and was stable across 100 simulations, varying by only ~ 0.2%, as has been previously reported (eTable 5) [18].

© 2025 Krauland MG et al. *JAMA Network Open*.

eTable 5. Influenza Model Immunity From Prior Year Infection

Age group	Percent Immune	Number with Prior Immunity (total in age group)
0-1	0	0 (24,749)
2	12.54	1,635 (13,039)
3-4	25.08	6,327 (25,228)
5-17	18.48	34,465 (186,501)
18-49	13.32	67,356 (505,676)
50-64	16.08	41,252 (256,540)
>=65	4.32	8,941 (206,962)

6. Vaccination

Vaccination was done by household. Households were chosen to vaccinate and agents in those households were vaccinated at rates that result in rate by age-group similar to CDC reported vaccine uptake for 2019-20 rates: age 0.5-17, 63.8%; age 18-49, 38.4%; age 50-64, 50.6%; age 65+, 69.8% (CDC reported rates, https://www.cdc.gov/flu/fluvaxview/coverage-1920estimates.htm). Agents under the age of 6 months are not vaccinated. Vaccination is distributed uniformly over 45 days beginning on September 20 of the simulation. Vaccinated agents receive immunity 14 days after vaccination. Vaccination and prior immunity are distributed separately in the population. Agents who have reduced susceptibility due to prior immunity receive an addition reduction in susceptibility due to vaccination at a reduced rate described by: current susceptibility - (current susceptibility * vaccine effectiveness). While the agents are randomly chosen for vaccination in an individual run, the desired number of agents is stable over 100 runs (varied by ~0.08% in the base scenario) [18]. The overall population vaccination rate was ~51%. For simulations that increased or decreased vaccination uptake, individual age group specific rates were increased or decreased in increments of 5% (eTable 6).

eTable 6. Percentage of Agents Vaccinated by Age in Base Scenario and in Decreased and Increased Vaccination Scenarios

Age group	Base	Decreased percent vaccinated						Increased percent vaccinated			
		5%[1]	10%	15%	20%	25%	30%	5%[2]	10%	15%	20%
0.5-17	63.80%	58.80%	53.80%	48.80%	43.80%	38.80%	33.80%	68.80%	73.80%	78.80%	83.80%
18-49	38.40%	33.40%	28.40%	23.40%	18.40%	13.40%	8.40%	43.40%	48.40%	53.40%	58.40%
50-64	50.60%	45.60%	40.60%	35.60%	30.60%	25.60%	20.60%	55.60%	60.60%	65.60%	70.60%
65+	69.80%	64.80%	59.80%	54.80%	49.80%	44.80%	39.80%	74.80%	79.80%	84.80%	89.80%
Total	51.04%	46.07%%	41.09%	36.12%	31.15%	26.17%	21.72%	56.02%	60.99%	65.97%	70.95%

[1] Percent decrease in number vaccinated
[2] Percent increase in number vaccinated

© 2025 Krauland MG et al. *JAMA Network Open*.

7. Sensitivity Analyses

We performed limited sensitivity analyses on several important simulation parameters (eTable 7). All simulations used the same model as described in Section 3, with noted changes to the applicable parameters. All scenarios were run with 100 iterations and means are reported.

eTable 7. Simulation Parameters Used for Sensitivity Analyses

Parameter	Value Range
FRED transmissibility	0.6 to 0.85 by 0.1
Percent of cases asymptomatic in 5-17 age group	25 to 100 by 5
Percent of cases asymptomatic in 18-49 age group	25 to 100 by 5
Percent of cases asymptomatic in 50-64 age group	25 to 100 by 5
Length of asymptomatic infectious period	lognormal mean 1 to 8, transmissibility parameter 0.60 to 0.85
Relative transmissibility of asymptomatic cases	25 to 100% by 5
Presence of immunity from prior infection	Without prior immunity compared to with prior immunity
Timing of vaccination	15 days before base case time of September 20 to February 2 by 15 day increments

a. FRED transmissibility

Increasing the FRED transmissibility parameter produced a nearly linear increase in mean cases (total population size 1,218,695) (eFigure 4).

© 2025 Krauland MG et al. *JAMA Network Open*.

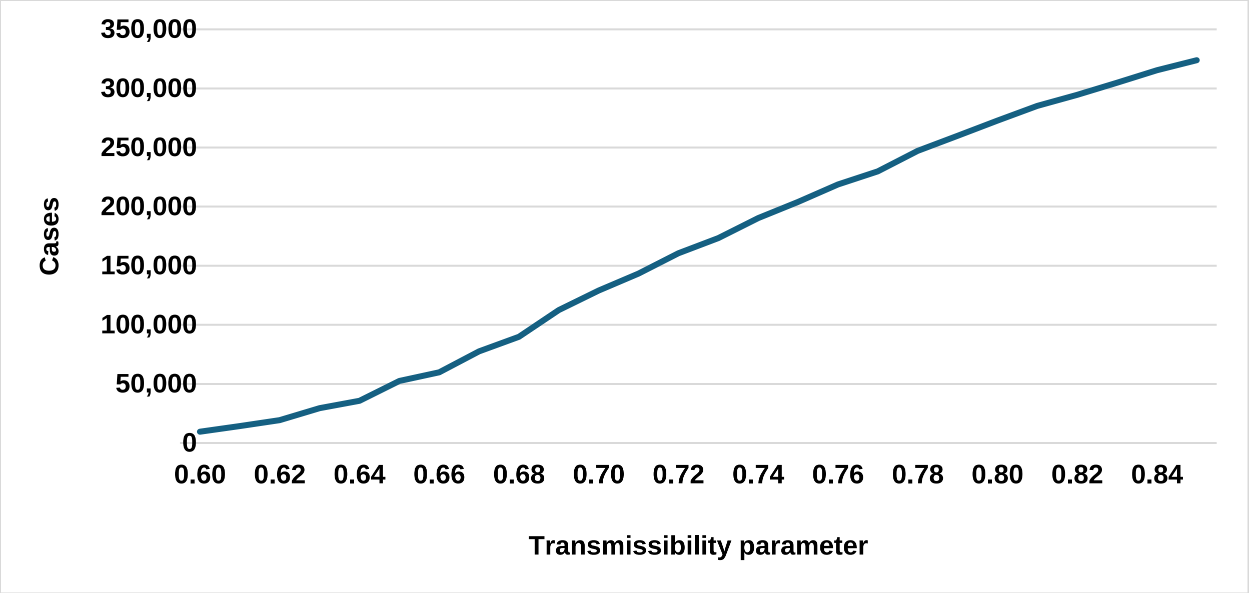

eFigure 4. Total Cases With Varied Transmissibility Parameter

b. Percent of cases asymptomatic by age group

The impact of changing the asymptomatic proportion of total cases differed by age group. Main simulations used 25% asymptomatic for all age groups. Asymptomatic agents are infectious at half the level of symptomatic cases.

As the percent of 5-17 year olds who were asymptomatic in the model increased from 5 to 100, total cases decreased (eFigure 5). Total cases decreased with an increase in percent of cases in 18-49 age group being asymptomatic but the model is less sensitive to this parameter than in the 5-17 age group. The model was relatively insensitive to the percent of agents in the 54-60 age group being asymptomatic. These results reflect that the 5-17 age group is a driver of influenza infections in the model.

© 2025 Krauland MG et al. *JAMA Network Open*.

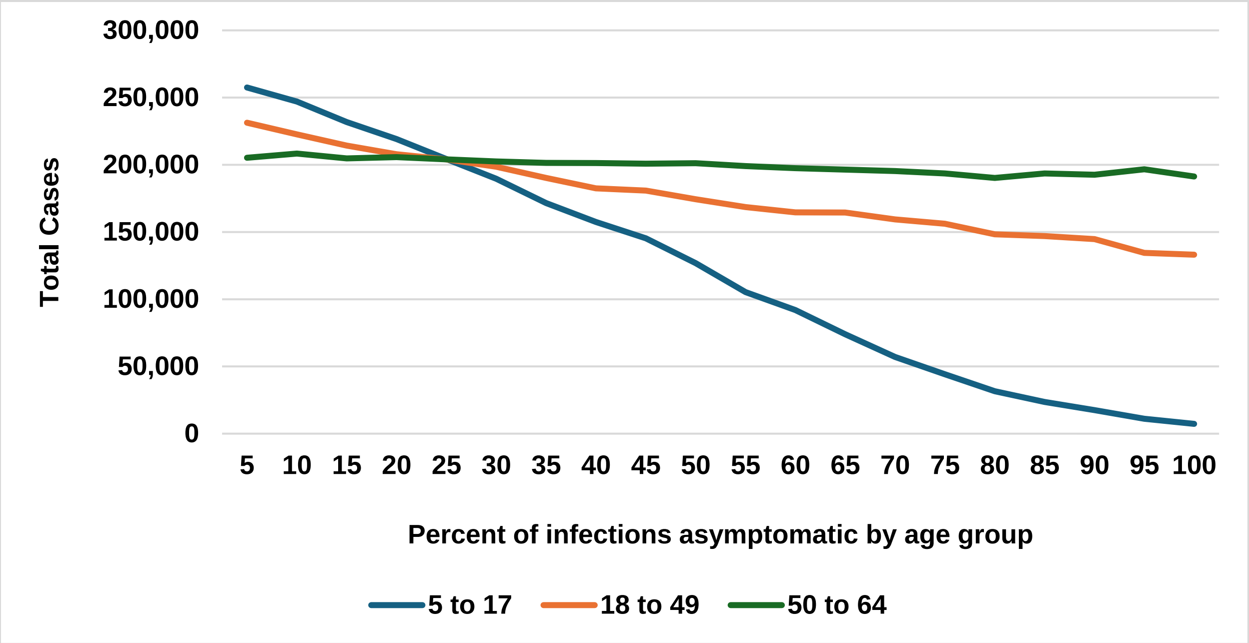

eFigure 5. Total Cases With Varied Percentage of Infections Being Asymptomatic in Different Age Groups

c. Length of asymptomatic infectious period

In the base model, asymptomatic infections are half as transmissible as symptomatic infections and 25% of infected agents are asymptomatic. The main model draws values for length of asymptomatic period for individual agents from a lognormal distribution with $\mu = 4$, $\sigma = 1.5$. Asymptomatic agents are expected to be infectious for a shorter time than symptomatic agents, however there is little data to support that parameter.

The model is sensitive to the length of the asymptomatic infectious state (eFigure 6). At a medium value for FRED transmissibility (0.75) when μ drops to 1, cases are reduced by 80% compared to $\mu = 5$. When μ increases from 5 to 8, total cases increase by ~ 40%. At lower values for the transmissibility parameter, lengths for the asymptomatic state of less than 4-5 days drastically reduces total infections. Presumably this effect is due to reduction in the effective reproductive rate, which is lower at lower values of transmissibility. The ~R_t was < 1 to slightly above 1 for shorter lengths of the asymptomatic state for both FRED transmissibilities of 0.6 and 0.65.
At all values of transmissibility, the effect of increasing length becomes essentially linear at higher state lengths.

© 2025 Krauland MG et al. *JAMA Network Open*.

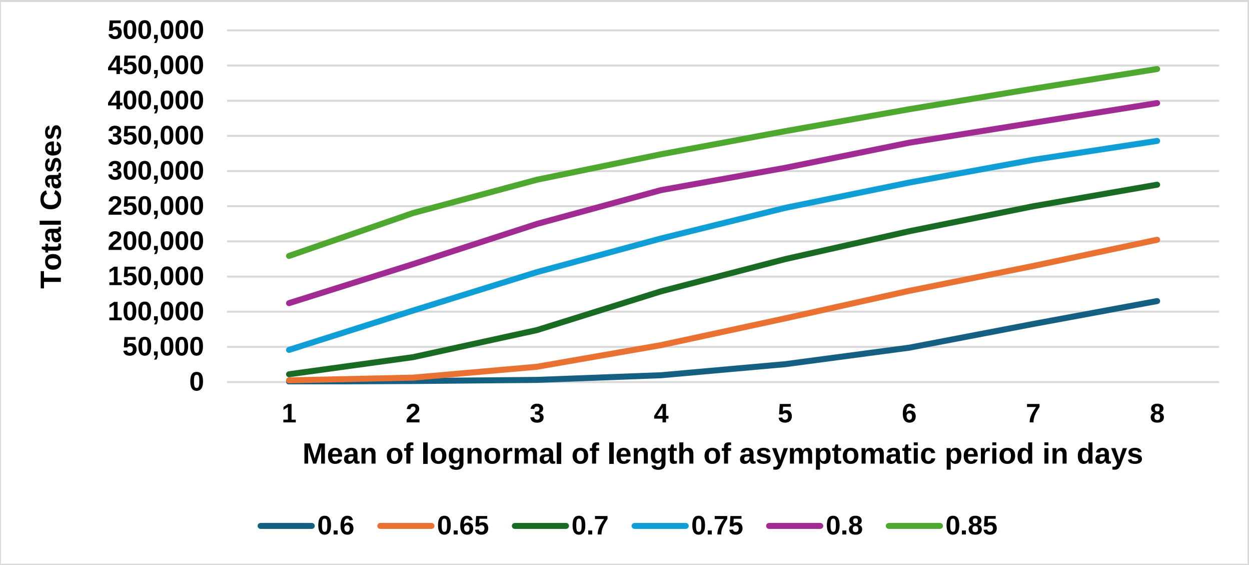

eFigure 6. Effect on Total Cases of Changes in Length in the Infectious Asymptomatic State Over Different Values for FRED Transmissibility Parameter

d. Relative transmissibility of asymptomatic cases

Increased relative infectiousness of asymptomatic cases has an almost linear effect on total cases for all tested values of the FRED transmissibility parameter (eFigure 7).

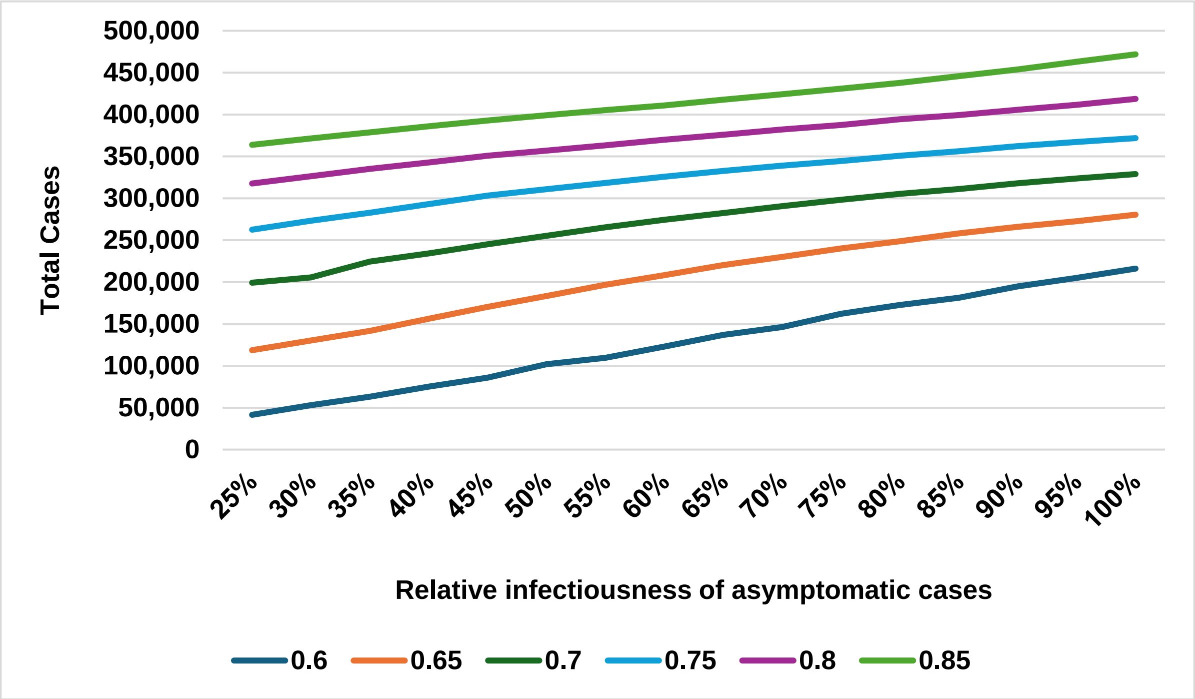

eFigure 7. Impact of Varying the Relative Infectiousness of Asymptomatic Cases (50% in base model)

© 2025 Krauland MG et al. *JAMA Network Open*.

e. Presence of immunity from prior infection

The inclusion of immunity caused by prior infection caused a decrease in total cases for all tested values of the FRED transmissibility parameter (eTable 8). The level of decrease caused by prior immunity decreased as the transmissibility increased.

eTable 8. Percent Difference in Total Cases Between Scenarios With and Without Immunity From Prior Infection

FRED transmissibility parameter value	Percent difference in total cases*
0.6	16.18%
0.65	12.76%
0.7	13.10%
0.75	13.89%
0.8	13.47%
0.85	11.43%

*Calculated as (total cases with no prior immunity – total cases with prior immunity) / total cases with no prior immunity. Means of 100 iterations are reported.

f. Sensitivity analysis of vaccination timing

Vaccination as reported by the CDC occurs mainly between epi week 31 and epi week 45, although a small addition number of vaccinations occurs later in the influenza season (eFigure 8). To determine how sensitive the model is to the timing of vaccination we ran identical models with the same vaccination mode of distribution as in the models described in this study and whose only difference was the start date for vaccination (eTable 9). We used an ~R_t of 1.81 and a VE of 40% and varied timing in increments of 15 days. We used total cases compared to the base case of September 20 as start of vaccination as the outcome variable. In this model, transmission peaks in mid-January.

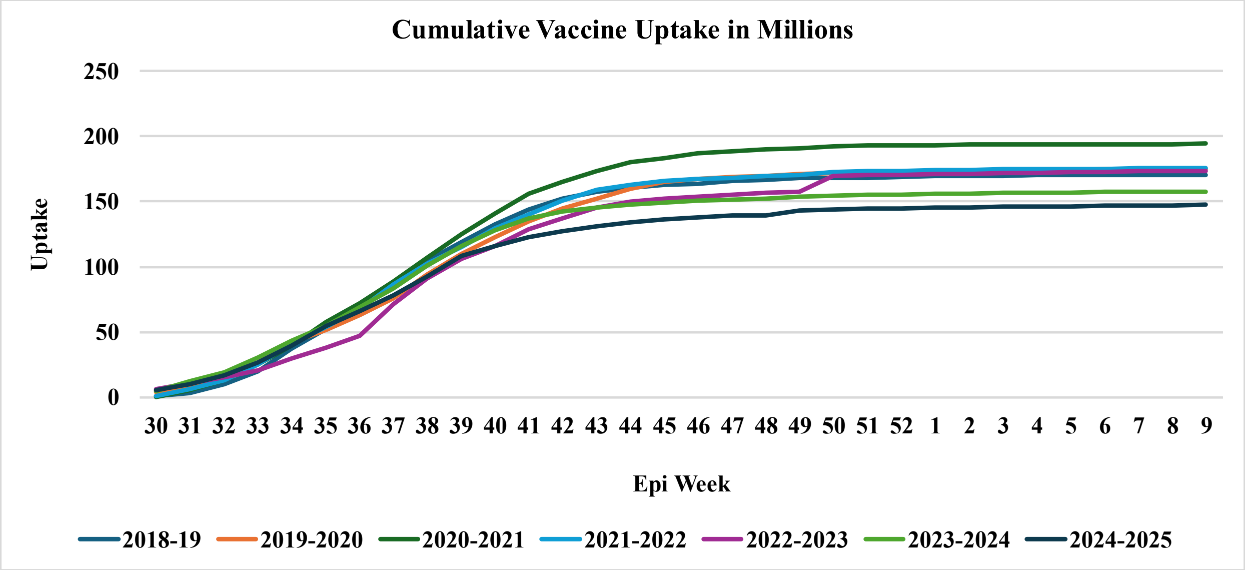

eFigure 8. Cumulative Vaccination Uptake as Reported by FluVaxView (https://www.cdc.gov/fluvaxview/dashboard/vaccine-doses-distributed.html).

© 2025 Krauland MG et al. *JAMA Network Open*.

Beginning vaccination 15 days earlier had a negligible effect on total cases (less than a 1% difference in total cases). Delaying vaccination by multiples of 15 days resulted in increasing case burdens until vaccination began at the end of December, at which time cases plateaued (eTable 9). We also tested normal versus uniform distribution of vaccination doses using the base case of September 20 for vaccination start. Using a normal distribution with mean of 22 and sigma of 10 resulted in a difference of 0.15% from the uniform distribution (1, 45) in a model that was otherwise identical.

eTable 9. Sensitivity Analysis of Timing of Vaccine Distribution

The base case started vaccination on September 20.

Timing difference	Date	Percent difference in case burden from base case
15 days earlier	September 5	- <1%
15 days later	October 5	6.45%
30 days later	October 20	23.02%
45 days later	November 4	42.63%
60 days later	November 19	61.68%
75 days later	December 4	75.27%
90 days later	December 19	82.53%
105 days later	January 3	85.03%
120 days later	January 18	85.72%

8. Populations with different demographics

To determine if our results were similar in populations with different demographics, specifically different age structure in the population, we repeated selected scenarios in 4 additional counties, one rural and 3 with large cities with different age structures (eTables 10 and11). The ratio of AR in unvaccinated to vaccinated varied slightly by county but behaved in a manner consistent with results for Allegheny County (eTable 11). Since vaccination uptake is by age-group in the simulation, the percent vaccinated varied by county based on the specific age structure but only by a small amount (49.9 to 51.8% of total population). In these results, a shift to more agents in the younger age groups was accompanied by a decrease in the ratio of indirect to direct benefit, indicating an increase in the indirect benefit of vaccination in a younger population relative to an older population.

© 2025 Krauland MG et al. *JAMA Network Open*.

eTable 10. Characteristics of Additional Simulation Counties

	Allegheny County, PA	Jefferson County, PA	Philadelphia County, PA	Bexar County, TX	Salt Lake City County, UT
Total Population	1,218,695	45,318	1,508,447	1,706,302	1,023,866
Percent of Agents by Age Group					
0-4	5.17%	5.56%	7.06%	7.81%	8.80%
5-17	15.30%	16.06%	18.08%	20.87%	21.66%
18-49	41.49%	39.01%	45.78%	44.80%	46.04%
50-64	21.05%	20.96%	16.58%	16.18%	15.00%
65-100	16.98%	18.41%	12.51%	10.33%	8.50%
Number of Households	549,592	18,997	622,421	623,845	349,701
Household Size	Percent of Agents in Households of Specified Size				
1	34.83%	28.63%	33.92%	25.48%	22.29%
2	33.89%	35.98%	29.10%	29.43%	29.40%
3	14.87%	15.77%	16.05%	16.74%	15.95%
4	10.40%	12.04%	10.67%	14.21%	14.43%
5	4.11%	4.82%	5.67%	7.89%	8.89%
6	1.30%	1.68%	2.58%	3.40%	4.96%
7	0.42%	0.76%	1.28%	1.44%	2.29%

As explained in Section S3, FRED uses a combination of disease natural history, population characteristics, population mixing rates and a transmissibility parameter to generate disease transmission. Because population characteristics such as demographics and household influence transmission rates, the attack rate differs slightly in counties with difference population makeup when no other parameters are changed (eTable 11).

eTable 11. Relationship of FRED Transmissibility Parameter to Attack Rate (AR) in Counties With Different Demographics in the Influenza Model With Only Difference Being Either Transmissibility Parameter or County Structure

Transmissibility parameter	Allegheny	Jefferson	Philadelphia	Bexar	Salt Lake City
0.6	21.16[1] (0.89)[2]	22.72 (1.29)	24.71 (0.35)	30.96 (0.24)	33.78 (0.49)
0.65	26.02 (0.25)	28.19 (1.18)	28.73 (0.41)	35.39 (0.43)	38.60 (0.36)
0.7	30.39 (0.23)	33.40 (0.96)	32.83 (0.30)	39.75 (0.25)	44.02 (0.53)
0.75	35.03 (0.25)	38.11 (0.86)	37.57 (0.51)	44.79 (0.43)	50.07 (0.71)
0.8	40.11 (0.54)	42.68 (0.70)	42.58 (0.62)	50.16 (0.64)	55.41 (0.48)
0.85	45.49 (0.67)	47.06 (0.68)	47.55 (0.48)	55.11 (0.50)	59.90 (0.29)

[1]Attack rate as percent
[2] Standard deviation

© 2025 Krauland MG et al. *JAMA Network Open*.

eTable 12. Ratio of Attack Rate (AR) in Unvaccinated to AR in Vaccinated in Counties With Different Demographics Over Varied Base ARs With No Vaccination and Varied Values of Vaccine Efficacy, Using Base Influenza Model

FRED transmissibility parameter (~R_t)[1]	Vaccine Efficacy	Allegheny County, PA (51.0)[2]	Jefferson County, PA (51.8)	Philadelphia County, PA (50.1)	Salt Lake City, UT (49.9)	Bexar County, TX (50.4)
0.6 (1.33)	30%	1.22	1.35	1.16	1.15	1.15
	40%	1.29	1.47	1.19	1.18	1.17
	50%	1.49	1.69	1.28	1.24	1.25
	60%	1.97	2.26	1.50	1.39	1.43
	70%	2.66	2.81	1.99	1.70	1.82
0.65 (1.33)	30%	1.29	1.38	1.24	1.23	1.22
	40%	1.31	1.44	1.27	1.26	1.25
	50%	1.39	1.57	1.31	1.31	1.29
	60%	1.61	1.82	1.42	1.39	1.38
	70%	2.10	2.47	1.67	1.55	1.58
0.70 (1.43)	30%	1.39	1.45	1.32	1.31	1.30
	40%	1.40	1.49	1.35	1.33	1.33
	50%	1.44	1.59	1.39	1.38	1.37
	60%	1.54	1.77	1.47	1.46	1.44
	70%	1.79	2.16	1.59	1.56	1.55
0.75 (1.88)	30%	1.48	1.52	1.41	1.39	1.38
	40%	1.50	1.57	1.43	1.41	1.40
	50%	1.54	1.63	1.47	1.45	1.44
	60%	1.60	1.77	1.54	1.51	1.51
	70%	1.73	2.06	1.65	1.62	1.61
0.8 (1.96)	30%	1.58	1.62	1.49	1.46	1.45
	40%	1.60	1.66	1.51	1.49	1.47
	50%	1.63	1.72	1.55	1.52	1.51
	60%	1.70	1.83	1.61	1.58	1.57
	70%	1.80	2.02	1.72	1.68	1.67
0.85 (1.98)	30%	1.68	1.69	1.58	1.53	1.53
	40%	1.70	1.74	1.60	1.56	1.55
	50%	1.72	1.79	1.63	1.61	1.58
	60%	1.78	1.89	1.68	1.66	1.63
	70%	1.89	2.03	1.78	1.75	1.72

[1] ~R_t as output from FRED simulations in model with no vaccination with the indicated FRED transmissibility parameter
[2] Percent vaccinated, varies due to differences in age group composition of counties.

© 2025 Krauland MG et al. *JAMA Network Open*.

9. Averted Burden Additional Results

eTable 13. Percent Reduction in Influenza Burden Due to Vaccination, With Varied Transmissibility of the Strain (~R_t) and Effectiveness of the Vaccine

	~R_t[1]					
Vaccine Effectiveness[2]	1.33	1.43[3]	1.81[4]	1.88[5]	1.96	1.98
40%	55.7 (6.0)[6]	41.5 (3.4)	34.3 (1.5)	32.9 (0.9)	34.3 (1.6)	36.5 (1.6)
45%	64.2 (6.7)	48.4 (3.4)	38.9 (2.0)	35.8 (0.9)	36.4 (1.5)	38.3 (1.7)
50%	72.8 (6.8)	56.1 (5.0)	43.6 (2.0)	39.2 (1.1)	39.0 (1.5)	40.2 (1.6)
55%	81.2 (5.8)	62.4 (3.6)	50.3 (2.8)	43.3 (1.3)	41.8 (1.5)	42.5 (1.6)
60%	88.6 (4.2)	70.3 (4.0)	56.6 (4.1)	48.1 (1.8)	45.1 (1.4)	45.1 (1.4)

[1] Approximate R_t produced by model
[2] Implemented as decrease in susceptibility to infection in the model
[3] Similar to low transmission for seasonal influenza
[4] Similar to medium transmission for seasonal influenza
[5] Similar to high transmission for seasonal influenza
[6] Mean (standard deviation) of 100 simulations

© 2025 Krauland MG et al. *JAMA Network Open*.

eTable 14. Percentage of Total Cases Averted by Vaccination With Varied Vaccine Uptake

Vaccine efficacy[1]	Percent vaccinated[2]	~R_t[3]					
		1.33	1.43	1.81	1.88	1.96	1.98
40%	22%	24.5 (9.0)[4]	16.8 (2.6)	15.0 (2.4)	15.5 (1.5)	17.0 (1.7)	18.4 (1.8)
	26%	29.2 (9.3)	20.6 (3.2)	18.1 (1.5)	18.5 (1.4)	20.3 (1.8)	21.7 (1.6)
	31%	34.5 (8.2)	25.1 (6.9)	21.1 (1.3)	21.6 (1.3)	23.4 (1.8)	25.2 (1.7)
	36%	41.3 (10.0)	29.5 (6.8)	24.7 (1.6)	24.6 (1.3)	26.3 (1.9)	28.3 (1.7)
	41%	46.7 (8.6)	33.6 (4.6)	27.9 (1.8)	27.5 (1.3)	29.3 (2.0)	31.2 (1.9)
	46%	51.6 (8.3)	37.7 (3.8)	31.2 (2.2)	30.2 (1.2)	31.8 (1.7)	33.9 (1.8)
	51%[5]	55.7 (6.0)	41.5 (3.4)	34.3 (1.5)	32.9 (0.9)	34.3 (1.6)	36.5 (1.6)
	56%	62.5 (6.9)	47.2 (3.9)	38.0 (1.7)	35.7 (1.1)	36.6 (1.5)	38.7 (1.8)
	61%	67.1 (7.3)	50.6 (5.2)	41.1 (2.2)	38.4 (1.2)	39.0 (1.5)	40.8 (1.6)
	66%	71.3 (7.1)	54.9 (5.8)	44.7 (2.2)	41.1 (1.4)	41.4 (1.6)	43.0 (1.7)
	71%	77.5 (7.4)	57.8 (2.9)	48.0 (2.1)	43.8 (1.3)	43.6 (1.5)	45.1 (1.6)
50%	22%	32.9 (8.7)	21.6 (3.0)	18.1 (2.3)	18.0 (1.4)	19.2 (1.7)	20.8 (1.6)
	26%	39.2 (10.0)	26.4 (3.6)	21.8 (1.6)	21.4 (1.2)	23.0 (1.8)	24.4 (1.7)
	31%	45.7 (8.3)	32.6 (6.8)	25.8 (1.4)	25.1 (1.2)	26.4 (1.8)	28.1 (1.7)
	36%	53.8 (8.3)	37.9 (4.1)	30.8 (3.3)	28.7 (1.2)	29.7 (1.7)	31.4 (1.8)
	41%	61.0 (8.0)	43.3 (4.3)	35.0 (3.4)	32.4 (1.3)	33.0 (1.6)	34.7 (1.8)
	46%	66.9 (7.5)	49.4 (3.8)	39.1 (1.9)	35.8 (1.4)	36.0 (1.5)	37.5 (1.7)
	51%	72.8 (6.8)	56.1 (5.0)	43.6 (2.0)	39.2 (1.1)	39.0 (1.5)	40.2 (1.6)
	56%	81.1 (6.5)	61.0 (4.0)	49.1 (2.9)	42.9 (1.4)	41.8 (1.3)	42.8 (1.5)
	61%	84.1 (5.8)	65.8 (4.1)	53.4 (2.6)	46.7 (1.7)	44.9 (1.4)	45.5 (1.4)
	66%	90.4 (4.8)	70.6 (4.1)	58.5 (2.9)	51.0 (2.0)	47.8 (1.5)	48.0 (1.3)
	71%	92.6 (3.6)	76.8 (5.6)	62.8 (2.4)	55.1 (2.0)	51.1 (1.9)	50.4 (1.1)
60%	22%	41.4 (11.4)	26.3 (2.9)	21.9 (2.0)	20.9 (1.4)	22.0 (1.7)	23.4 (1.6)
	26%	49.3 (8.5)	32.9 (4.1)	26.3 (1.8)	25.0 (1.2)	26.0 (1.8)	27.4 (1.7)
	31%	58.5 (9.5)	41.0 (5.4)	31.7 (1.7)	29.4 (1.5)	30.0 (1.7)	31.5 (1.7)
	36%	67.0 (8.3)	49.1 (4.9)	38.7 (4.4)	33.7 (1.3)	33.8 (1.6)	35.0 (1.7)
	41%	75.1 (6.0)	56.2 (5.1)	44.0 (4.5)	38.5 (1.5)	37.6 (1.6)	38.6 (1.6)
	46%	82.1 (5.7)	63.3 (4.2)	49.6 (2.7)	43.3 (2.1)	41.4 (1.4)	41.9 (1.7)
	51%	88.6 (4.2)	70.3 (4.0)	56.6 (4.1)	48.1 (1.8)	45.1 (1.4)	45.1 (1.4)
	56%	94.2 (3.1)	77.9 (5.1)	63.4 (3.1)	53.7 (2.0)	49.6 (2.4)	48.3 (1.4)
	61%	96.0 (1.9)	84.7 (4.2)	68.6 (2.4)	59.0 (2.2)	53.1 (1.7)	51.5 (1.3)
	66%	98.0 (1.3)	90.3 (3.7)	75.1 (3.7)	64.4 (2.3)	58.0 (1.8)	54.9 (1.5)
	71%	99.0 (0.8)	94.1 (2.7)	80.7 (3.7)	69.0 (1.7)	62.8 (2.0)	58.7 (1.4)

[1] Decrease in susceptibility to infection in the model; [2] Percent of the population vaccinated
[3] Approximate R_t produced by model with no vaccination; [4] Mean of 100 simulations (standard deviation)
[5] Percent vaccinated in main model scenarios

© 2025 Krauland MG et al. *JAMA Network Open*.

eTable 15. Direct and Indirect Benefits of Vaccination in High Transmission Scenarios

Vaccine effectiveness	Attack Rate in unvaccinated	Attack Rate in vaccinated	Percent reduction in total cases in population[1]	Percent reduction in cases in unvaccinated[2]	Percent reduction in cases in vaccinated[3]	Ratio Attack Rate in unvaccinated / Attack Rate in vaccinated
No Vaccination	51.13% (0.47%)[4] (~R_t 2.01)[5]	-	-	-	-	-
0.3	44.59% (0.26%)	23.67% (0.42%)	66.33%	12.79%	53.70%	1.88
0.35	43.58% (0.32%)	22.92% (0.57%)	64.61%	14.77%	55.17%	1.90
0.4	42.42% (0.37%)	22.05% (0.58%)	62.63%	17.03%	56.88%	1.92
0.45	41.36% (0.40%)	21.29% (0.67%)	60.85%	19.11%	58.37%	1.94
0.5	40.17% (0.44%)	20.42% (0.63%)	58.85%	21.43%	60.06%	1.97
0.55	38.96% (0.39%)	19.61% (0.65%)	56.88%	23.80%	61.65%	1.99
0.6	37.69% (0.42%)	18.78% (0.67%)	54.84%	26.28%	63.27%	2.01
0.65	36.16% (0.34%)	17.74% (0.58%)	52.34%	29.27%	65.30%	2.04
0.7	34.55% (0.28%)	16.63% (0.52%)	49.69%	32.42%	67.47%	2.08
No Vaccination	85.39% (0.07%) (~R_t 3.92)	-	-	-	-	-
0.3	84.73% (0.11%)	39.43% (0.41%)	72.15%	0.78%	53.82%	2.15
0.35	84.40% (0.11%)	38.98% (0.42%)	71.69%	1.17%	54.35%	2.17
0.4	84.01% (0.12%)	38.41% (0.39%)	71.12%	1.62%	55.02%	2.19
0.45	83.55% (0.11%)	37.79% (0.40%)	70.49%	2.16%	55.74%	2.21
0.5	82.99% (0.12%)	36.88% (0.37%)	69.62%	2.82%	56.81%	2.25
0.55	82.35% (0.13%)	35.88% (0.40%)	68.66%	3.56%	57.99%	2.30
0.6	81.60% (0.13%)	34.65% (0.39%)	67.49%	4.45%	59.42%	2.35
0.65	80.69% (0.13%)	33.15% (0.38%)	66.07%	5.51%	61.18%	2.43
0.7	79.63% (0.15%)	31.41% (0.36%)	64.43%	6.75%	63.21%	2.54
No Vaccination	93.64% (0.04%) (~R_t 4.99)	-	-	-	-	-
0.3	95.95% (0.35%)	41.26% (0.60%)	72.66%	-2.46%[6]	55.93%	2.33
0.35	95.75% (0.31%)	41.15% (0.55%)	72.49%	-2.25%	56.05%	2.33
0.4	95.51% (0.32%)	40.92% (0.55%)	72.24%	-2.00%	56.30%	2.33
0.45	95.20% (0.32%)	40.68% (0.56%)	71.95%	-1.66%	56.55%	2.34
0.5	94.87% (0.33%)	40.21% (0.58%)	71.52%	-1.32%	57.06%	2.36
0.55	94.50% (0.30%)	39.58% (0.59%)	70.99%	-0.92%	57.73%	2.39
0.6	94.07% (0.30%)	38.76% (0.58%)	70.31%	-0.46%	58.61%	2.43
0.65	93.58% (0.30%)	37.61% (0.57%)	69.43%	0.06%	59.84%	2.49
0.7	92.95% (0.27%)	36.25% (0.58%)	68.35%	0.74%	61.28%	2.56
No Vaccination	96.58% (0.03%) (~R_t 5.04)	-	-	-	-	-
0.3	102.31% (0.53%)[7]	40.61% (0.63%)	73.32%	-5.93%	57.95%	2.52

© 2025 Krauland MG et al. *JAMA Network Open*.

0.35	102.18% (0.50%)	40.59% (0.60%)	73.25%	-5.80%	57.98%	2.52
0.4	101.97% (0.52%)	40.57% (0.61%)	73.14%	-5.59%	57.99%	2.51
0.45	101.74% (0.50%)	40.45% (0.63%)	72.95%	-5.34%	58.11%	2.51
0.5	101.48% (0.46%)	40.24% (0.53%)	72.71%	-5.08%	58.33%	2.52
0.55	101.22% (0.43%)	39.86% (0.53%)	72.38%	-4.81%	58.73%	2.54
0.6	100.88% (0.42%)	39.35% (0.52%)	71.94%	-4.46%	59.26%	2.56
0.65	100.54% (0.46%)	38.67% (0.53%)	71.40%	-4.10%	59.96%	2.60
0.7	100.10% (0.46%)	37.71% (0.52%)	70.67%	-3.65%	60.96%	2.65

[1] Calculated as total cases with vaccination / total cases in base scenario that does not include vaccination
[2] Calculated as total cases in the unvaccinated portion of the population / (attack rate in unvaccinated model * number unvaccinated)
[3] Calculated as total cases in the vaccinated portion of the population / (attack rate in unvaccinated model * number vaccinated)
[4] Standard deviation
[5] ~R_t produced by FRED simulation with no vaccination
[6] The unvaccinated population may have a higher attack rate than the total population has in the absence of vaccination, resulting in a negative reduction
[7] Reinfections during the year due to waning of immunity result in a small number of agents being infected twice

To further explore the decrease in indirect benefit as transmissibility increased, we ran a sensitivity analysis with increasing transmissibility between ~R_t of 2.01 and 3.92. This model included vaccination at the base population level of ~51% and 40% VE. The indirect benefit decreased in a bimodal fashion with a relatively linear decrease which changed slope at ~ R_t of 2.97 (FRED transmissibility parameter of 1.5 in this model) (eFigure 9, eTable 16). This corresponds to a requirement for ~66% immunity to achieve herd immunity in this population (calculated as 1-1/R_0). Over the range of ~R_t tested, the direct benefit remained relatively stable (56.48% to 54.89%).

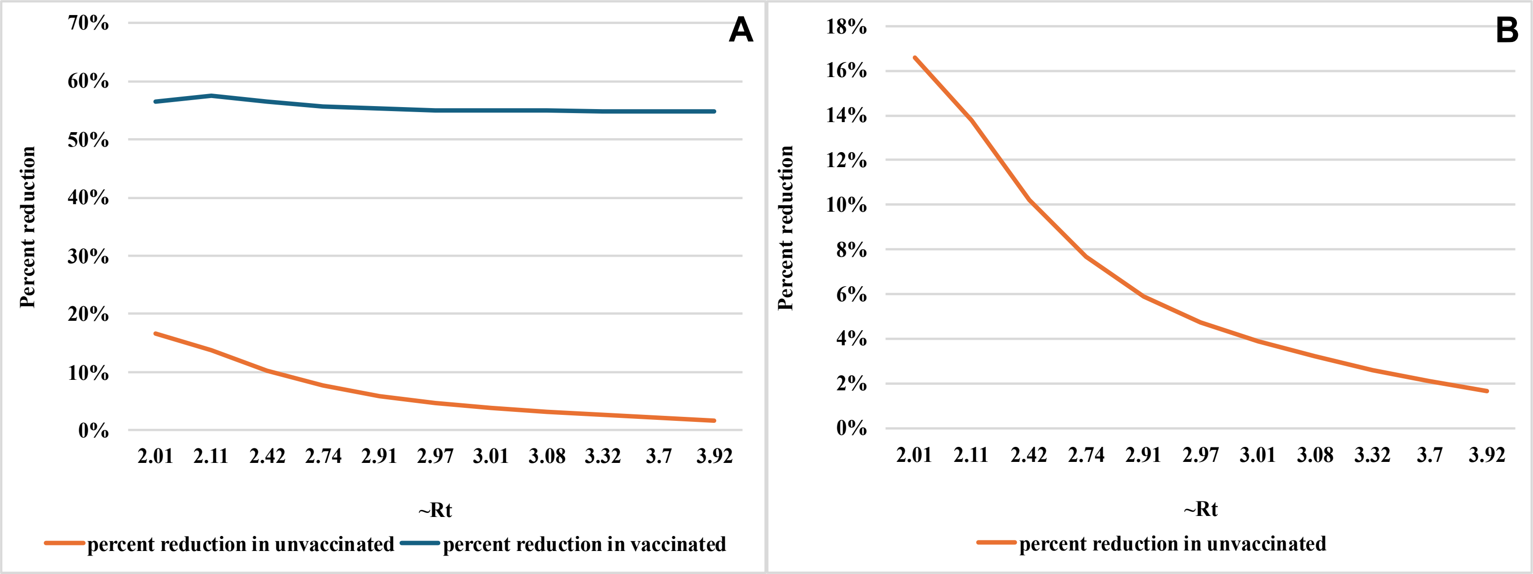

eFigure 9. Decrease in Indirect Benefit of Vaccination as ~R_t Increases From 2.01 to 3.92
A, direct (vaccinated) and indirect (unvaccinated) benefit; B, indirect (unvaccinated) benefit.

© 2025 Krauland MG et al. *JAMA Network Open*.

eTable 16. Relationship of ~R_t to Change in Amount of Direct and Indirect Benefit of Vaccination

~R_t in an unvaccinated population (FRED transmissibility parameter)	Approximate Level of Immunity for Herd Immunity	Attack Rate with No Vaccination	Attack Rate in Unvaccinated	Attack Rate in Vaccinated	Percent reduction in Attack Rate in Unvaccinated[1]	Percent reduction in Attack Rate in Vaccinated[2]
2.01 (1.0)	50.25%[3]	621,907 (5,691)[4]	42.56% (0.38%)[5]	22.21% (0.66%)	16.60%	56.48%
2.11 (1.1)	52.61%	706,745 (2,451)	50.01% (0.28%)	24.69% (0.20%)	13.77%	57.43%
2.42 (1.2)	58.68%	773,732 (1,339)	57.01% (0.29%)	27.62% (0.15%)	10.20%	56.50%
2.74 (1.3)	63.50%	829,312 (1,211)	62.84% (0.17%)	30.14% (0.10%)	7.65%	55.71%
2.91 (1.4)	65.64%	875,299 (1,330)	67.59% (0.16%)	32.14% (0.18%)	5.89%	55.26%
2.97 (1.5)	66.33%	914,494 (1,337)	71.49% (0.14%)	33.73% (0.21%)	4.73%	55.05%
3.01 (1.6)	66.78%	947,905 (1,193)	74.76% (0.15%)	35.03% (0.24%)	3.88%	54.96%
3.08 (1.7)	67.53%	976,335 (1,188)	77.55% (0.17%)	36.13% (0.34%)	3.20%	54.90%
3.32 (1.8)	69.88%	1,001,048 (1,163)	80.01% (0.13%)	37.09% (0.34%)	2.60%	54.85%
3.70 (1.9)	72.97%	1,022,405 (883)	82.14% (0.11%)	37.90% (0.39%)	2.10%	54.83%
3.92 (2.0)	74.49%	1,041,031 (937)	84.00% (0.12%)	38.53% (0.41%)	1.67%	54.89%

[1] Calculated as total cases in the unvaccinated portion of the population / (attack rate in unvaccinated model * number unvaccinated)
[2] Calculated as total cases in the vaccinated portion of the population / (attack rate in unvaccinated model * number vaccinated)
[3] Calculated as 1-(1/R_0)
[4]Standard deviation of 100 model iterations
[5] Standard deviation of 100 model iterations

© 2025 Krauland MG et al. *JAMA Network Open*.

eReferences 2

1. Ahmed F, Kim S, Nowalk MP, et al. Paid Leave and Access to Telework as Work Attendance Determinants during Acute Respiratory Illness, United States, 2017-2018. *Emerg Infect Dis.* 2020;26(1):26-33.
2. Cajka JC, Cooley, P. C., & Wheaton, W. D. *Attribute assignment to a synthetic population in support of agent-based disease modeling.* . RTI Press;2010.
3. Chasteen BM, Wheaton, W. D., Cooley, P. C., Ganapathi, L., & Wagener, D. K. *Including the group quarters population in the US synthesized population database.* RTI Press;2011. RTI Press Methods Report No. MR-0020-1102
4. Chrest DP, & Wheaton, W. D. . *Using geographic information systems to define and map commuting patterns as inputs to agent-based models.* RTI Press;2009.
5. Wheaton WD, Cajka, J. C., Chasteen, B. M., Wagener, D. K., Cooley, P. C., Ganapathi, L., Roberts, D. J., & Allpress, J. L. . *Synthesized population databases: A US geospatial database for agent-based models.* RTI Press;2009.
6. Kissler SM, Tedijanto C, Goldstein E, Grad YH, Lipsitch M. Projecting the transmission dynamics of SARS-CoV-2 through the postpandemic period. *Science.* 2020;368(6493):860-868.
7. Centers for Disease Control and Prevention. Estimated flu disease burden, by age group — United States, 2019-2020 flu season. https://www.cdc.gov/flu/about/burden/2019-2020.html. Accessed July 28, 2022.
8. Tsang TK, Perera R, Fang VJ, et al. Reconstructing antibody dynamics to estimate the risk of influenza virus infection. *Nat Commun.* 2022;13(1):1557.
9. Furuya-Kanamori L, Cox M, Milinovich GJ, Magalhaes RJ, Mackay IM, Yakob L. Heterogeneous and Dynamic Prevalence of Asymptomatic Influenza Virus Infections. *Emerg Infect Dis.* 2016;22(6):1052-1056.
10. Carrat F, Vergu E, Ferguson NM, et al. Time lines of infection and disease in human influenza: a review of volunteer challenge studies. *Am J Epidemiol.* 2008;167(7):775-785.
11. Cohen C, Kleynhans J, Moyes J, et al. Asymptomatic transmission and high community burden of seasonal influenza in an urban and a rural community in South Africa, 2017-18 (PHIRST): a population cohort study. *Lancet Glob Health.* 2021;9(6):e863-e874.
12. Centers for Disease Control and Prevention. Influenza (Flu), Vaccine Effectiveness Studies. https://www.cdc.gov/flu/vaccines-work/past-seasons-estimates.html. Published 2024. Accessed February 8, 2024.
13. Ferdinands JM, Fry AM, Reynolds S, et al. Intraseason waning of influenza vaccine protection: Evidence from the US Influenza Vaccine Effectiveness Network, 2011-12 through 2014-15. *Clin Infect Dis.* 2017;64(5):544-550.
14. Ferdinands JM, Gaglani M, Martin ET, et al. Waning Vaccine Effectiveness Against Influenza-Associated Hospitalizations Among Adults, 2015-2016 to 2018-2019, United States Hospitalized Adult Influenza Vaccine Effectiveness Network. *Clin Infect Dis.* 2021;73(4):726-729.

© 2025 Krauland MG et al. *JAMA Network Open*.

15. Lessler J, Reich NG, Brookmeyer R, Perl TM, Nelson KE, Cummings DA. Incubation periods of acute respiratory viral infections: a systematic review. *Lancet Infect Dis.* 2009;9(5):291-300.
16. Centers for Disease Control and Prevention. How Flu Spreads. https://www.cdc.gov/flu/spread/index.html. Published 2024. Accessed November 17, 2024, 2024.
17. Malosh RE, McGovern I, Monto AS. Influenza During the 2010-2020 Decade in the United States: Seasonal Outbreaks and Vaccine Interventions. *Clin Infect Dis.* 2023;76(3):540-549.
18. Krauland MG, Galloway DD, Raviotta JM, Zimmerman RK, Roberts MS. Impact of Low Rates of Influenza on Next-Season Influenza Infections. *Am J Prev Med.* 2022;62(4):503-510.
19. Krauland MG, Roberts MS. Modeling the Impact of COVID-19 Mitigation Strategies in Pennsylvania, USA. *MDM Policy Pract.* 2024;9(1):23814683241260744.
20. Krauland MG, Zimmerman RK, Williams KV, et al. Agent-based model of the impact of higher influenza vaccine efficacy on seasonal influenza burden. *Vaccine X.* 2023;13:100249.
21. Grefenstette JJ, Brown ST, Rosenfeld R, et al. FRED (a Framework for Reconstructing Epidemic Dynamics): an open-source software system for modeling infectious diseases and control strategies using census-based populations. *BMC Public Health.* 2013;13:940.

© 2025 Krauland MG et al. *JAMA Network Open*.
