# Supplementary material for: Estimated Burden of Influenza and Direct and Indirect Benefits of Influenza Vaccination
Source: JAMA Netw Open. 2025 Jul 16;8(7):e2521324. doi: 10.1001/jamanetworkopen.2025.21324 (PMC12268489; doi:10.1001/jamanetworkopen.2025.21324)
Supplement: Supplement 2. — Data Sharing Statement [file jamanetwopen-e2521324-s002.pdf]

Data Sharing Statement

Krauland. Averted Burden of Influenza and Direct and Indirect Benefits of Influenza Vaccination. *JAMA Netw Open*. Published July 16, 2025.
doi:10.1001/jamanetworkopen.2025.21324

Data
Data available: Yes
Data types: Data (not involving human participants)
How to access data: Data can be requested from mgk8@pitt.edu
When available: With publication

Supporting Documents
Document types: None

Additional Information
Who can access the data: anyone requesting data
Types of analyses: for any purpose
Mechanisms of data availability: upon request with no restrictions
Any additional restrictions: no restrictions
